# Unlocking binding properties of single-domain antibodies targeting the polymeric immunoglobulin receptor to enhance mucosal enrichment of IgG against respiratory syncytial virus

**DOI:** 10.3389/fimmu.2025.1739562

**Published:** 2026-02-09

**Authors:** Jia Liu, Jiwon Jung, Siqun Zhou, Shi-Juan Chen, Jiping Huang, Yinyan Tang, Karin Vroom, Soha Motlagh, Daoyang Chen, Huong Trinh, Peter Worthington, Daphne Y. Ma, Zhiyun Wen, Bin Luo, Daniela Bumbaca Yadav, Jia Yao Phuah, Zhifeng Chen, Kalpit A. Vora, Masahisa Handa

**Affiliations:** 1 Department of Discovery Biologics, Merck Research Laboratories (MRL), Merck & Co., Inc., Rahway, NJ, United States; 2 Department of Pharmacokinetics, Dynamics, Metabolism and Bioanalytics, Merck Research Laboratories (MRL), Merck & Co., Inc., Rahway, NJ, United States; 3 Department of Quantitative Biosciences, Merck Research Laboratories (MRL), Merck & Co., Inc., Rahway, NJ, United States; 4 Department of Infectious Diseases and Vaccines, Merck Research Laboratories (MRL), Merck & Co., Inc., Rahway, NJ, United States

**Keywords:** polymeric immunoglobulin receptor (pIgR), single-domain antibody (V_H_H), pH-dependent binding, respiratory syncytial virus (RSV), targeted mucosal delivery, bi-specific antibody

## Abstract

A major driver of mucosal immunity is immunoglobulin A (IgA) that can translocate across mucosal epithelial barriers to protect against various pathogens in luminal spaces of the human body. The transcytosis of IgA is primarily mediated by the polymeric Ig receptor (pIgR), which is highly expressed in mucosal tissues and selectively transports polymeric IgA, but not IgG. IgG has been the preferred immunoglobulin isotype for therapeutic development because of its well-characterized biological functions and established manufacturing processes. Efficient transport of IgG across the epithelium into mucosal spaces is a highly desirable feature in the development of IgG-based neutralizing antibodies targeting respiratory infections. To address this challenge, we report discovery and characterization of anti-pIgR variable single-domain antibodies (V_H_H) that facilitate pIgR-mediated transcytosis with efficiency comparable to dimeric IgA in epithelial cell models. Screening a panel of anti-pIgR V_H_H-Fc molecules targeting the same epitope bin revealed correlations between binding parameters (K_D_ and *k_off_*) and transcytosis activity. Notably, several antibodies with highly efficient transcytosis exhibited faster dissociation rates at acidic pH relative to neutral pH, suggesting the potential of pH-dependent binding as a factor to influence the transcytosis pathway of pIgR-bound antibodies. Building on these insights, we engineered a bispecific antibody (bsAb) by fusing an anti-pIgR V_H_H to the C-terminus of an IgG heavy chain targeting respiratory syncytial virus (RSV). This bsAb efficiently transcytosed across pIgR-expressing Madin-Darby canine kidney (MDCK) cells and a human airway mucosal barrier model, while fully retaining RSV binding and neutralization capabilities. Our study introduces a novel strategy to enhance the mucosal distribution of systemically administered biologics, with significant implications for the development of improved antibody therapeutics against mucosal pathogens.

## Introduction

The mucosal barrier constitutes a critical interface between the internal milieu and the external environment, serving as the first line of defense against harmful substances and microbial organisms (1). Numerous bacterial and viral agents colonize, replicate, and potentially initiate infection at mucosal surfaces, especially within the respiratory, gastrointestinal, and urogenital tracts (2). Therefore, effective immune surveillance and response at these sites are essential for preventing infection. A hallmark of mucosal immunity is the active transport of polymeric immunoglobulins, primarily dimeric IgA (dIgA) and pentameric IgM, across epithelial cells into mucosal secretions where they exert pathogen-neutralizing and immune exclusion functions to maintain mucosal homeostasis (1). IgA is the most abundant immunoglobulin in mucosal secretions and plays a pivotal role in immune defense by preventing microbial adherence and invasion (3). Clinically, selective IgA deficiency is characterized by recurrent mucosal infections, highlighting the importance of IgA in the mucosal defense system. Development of an IgA response has also been previously associated with better recovery in patients infected with RSV (4).

The transport of dIgA across the mucosal barriers is mediated by the polymeric immunoglobulin receptor (pIgR), a transmembrane receptor highly expressed on the basolateral surface of epithelial cells at mucosal sites (5). Human pIgR consists of a 620-residue ectodomain with five tandem Ig-like domains (D1-D5), a 23-residue transmembrane domain, and a 103-residue intracellular domain (6). dIgA is formed by joining two IgA monomers tail-to-tail through their heavy chain C-terminal tailpieces and a joining (J) chain (6). Recent structural studies revealed that pIgR binding to dIgA is initiated by non-covalent interactions between the pIgR D1 domain and the Cα2 domain of one IgA Fcα chain as well as the J-chain, followed by an extended conformational change that further stabilizes the complex through a disulfide bond between the pIgR D5 and the opposite IgA Fcα chain (7). The resulting pIgR-dIgA complex is internalized and trafficked to early endosomes in epithelial cells. Following a series of endocytic and transcytotic pathways, the complex is transported to the apical surface, where proteolytic cleavage releases the pIgR ectodomains as the secretory component (SC). dIgA remains covalently bound to SC forming secretory IgA (sIgA), which is protected from degradation in mucosal secretions (8–11). Despite the critical role of dIgA in mucosal immunity, its therapeutic application is limited by manufacturing challenges. Additionally, dIgA exists in heterogeneous polymeric forms with extensive glycosylation and exhibits a shorter *in vivo* half-life (12), necessitating more frequent dosing.

Given these limitations of dIgA, therapeutic development has currently focused on IgGs that have emerged as effective neutralizing antibodies in controlling respiratory viruses that continue to pose a major global health threat (13). Structurally, IgG consists of two main regions: the crystallizable fragment (Fc), which primarily mediates effector function, and the antigen-binding fragment (Fab), which confers high specificity and affinity to viral surface antigens, thereby blocking viral entry and subsequent infection (13). Additionally, optimization of the Fc domain enhances the regulation of effector function and prolongs serum half-life, bolstering IgG’s therapeutic potential (14). During the COVID-19 pandemic, neutralizing IgG antibodies have been essential for protecting vulnerable populations, such as the elderly and immunocompromised, with inadequate vaccine responses (15). Likewise, human respiratory syncytial virus (RSV) continues to threaten individuals across all ages, and prophylactic options for infants and young children are limited to maternal vaccination and passive immunization with RSV-neutralizing antibodies (16, 17). Several monoclonal IgG products have received FDA approval for this purpose. Recently, Beyfortus (nirsevimab) and Enflonsia (clesrovimab) have shown high efficacy in reducing RSV-related hospitalizations in vulnerable infants and children (18, 19). In this context, neutralizing antibodies, particularly IgG and IgG-based molecules, represent a promising tool to address the unmet medical needs associated with respiratory infectious diseases.

Although IgG is the most abundant immunoglobulin class in serum, it is present in mucosal secretions of the nasal and tracheobronchial regions at lower concentrations relative to total protein and IgA (8). For instance, monoclonal antibodies neutralizing SARS-CoV-2 achieved nasal lining fluid concentrations of only 5-10 µg/mL in healthy adults following a 3000 mg intravenous dose and showed acceptable efficacy in prophylactic settings (20). Hence, improving both the rate and magnitude of IgGs distribution in mucosal tissues is essential for advancing prophylactic and therapeutic interventions aimed at improving control of respiratory viral infections, especially amid emerging global public health threats. Multiple studies have reported that antibodies and peptides targeting pIgR can trigger its endocytosis on the basolateral surface of mucosal epithelial cells, followed by transport and release at the apical side (21–23). A previous study demonstrated that an anti-*Pseudomonas aeruginosa* IgG fused with a pIgR-binding peptide was able to transport and distribute effectively in the lung lumen, providing protection against acute *P. aeruginosa* pneumonia in mice (24). Another work described the use of pIgR-targeting antibody shuttles fused to angiotensin-converting enzyme 2 (ACE2) for mucosal enrichment of biologics against COVID-19 (25). However, further investigation is needed to translate pIgR targeting into an effective, broadly applicable modality for mucosal delivery of large molecules. The factors governing transcytosis activity of antibodies bound to pIgR also remain poorly characterized. In this study, we developed a diverse panel of anti-pIgR single-domain antibodies (V_H_H) that can effectively facilitate transcytosis in epithelial cells and act as a moiety fused to an anti-RSV IgG for the enhancement of IgG mucosal distribution in respiratory tracts. In addition, we investigated the relationship between the binding properties of anti-pIgR V_H_H molecules and the transcytosis activity in epithelial cells, aiming to provide in-depth insight into the engineering of biologics to enhance mucosal delivery.

## Results

### Generation and characterization of anti-pIgR single-domain (V_H_H) antibodies

**Figure 1:**
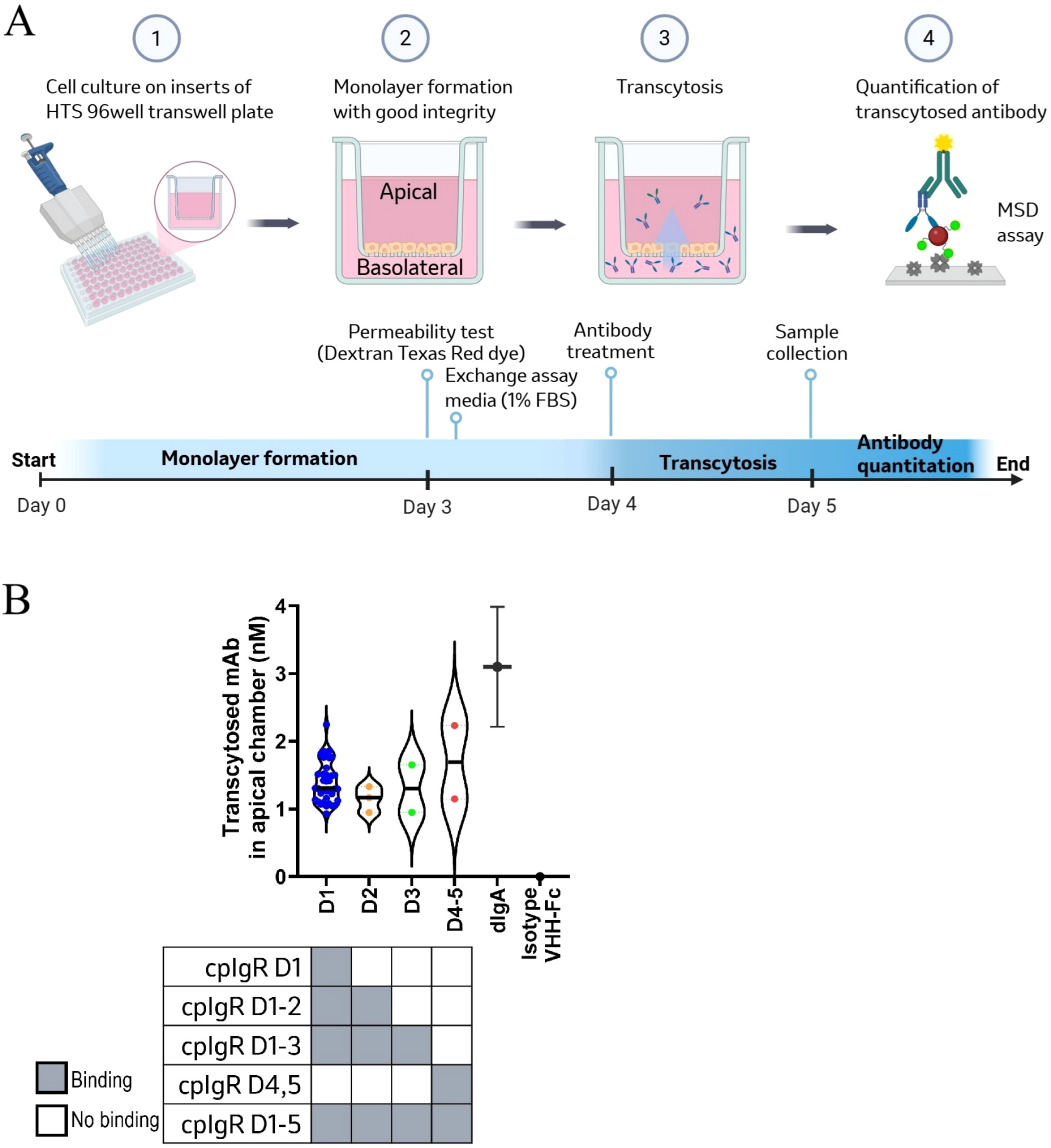
Transcytosis of anti-pIgR V_H_H-hFc molecules across pIgR-MDCK cells. **(A)** Schematic diagram of the epithelial model to evaluate transcytosis function of anti-pIgR V_H_H-hFc molecules. ① MDCK cells or cotton-rat pIgR (cpIgR) expressing MDCK cells were seeded on Transwell™ permeable inserts with 30,000 cells per well and cultured for 3 days to form a polarized epithelial monolayer. ② The integrity of monolayer culture was assessed on day 3 by measuring paracellular leakage of Dextran Texas Red. Flux range for intact cell monolayer was < 2%. After that, cells were washed with EMEM containing 1% FBS (assay media) and starved for overnight prior to treatment. ③ Antibody treatment was performed on day 4 by applying 100 nM of test or control molecules in the basolateral well and incubation for 24 hours at 37°C ④ On day 5, media was collected from the apical and basolateral chambers to assess the transcytosis activity of antibodies across epithelial monolayers. The concentrations of transcytosed antibodies in the apical media were quantitated by MSD assay. The diagram was created in BioRender. Liu, (J) (2025) https://BioRender.com/b3k2o5k. **(B)** Transcytosis activity is plotted vs. domain-level epitope mapping for anti-cpIgR V_H_H-hFc molecules. The top graph is a violin plot depicting the concentration of transcytosed V_H_H-hFc molecules in the apical chamber in 24-hour post treatment, measured in nanomolar (nM), across different domain binder groups labeled D1, D2, D3, and D4-5. Each color dot represents the average transcytosis value of three replicates for each antibody tested. A black bar within each violin indicates the median concentration for one group. dIgA was included in the transcytosis activity assessment as a positive control with deviation bars represent mean ± SD, and isotype V_H_H-hFc included as a negative control. Domain mapping results from SPR analysis are depicted in the bottom checkerboard diagram. Dark square indicates individual antibody group capable of binding to specific cpIgR domain constructs.

The cotton rat (*Sigmodon hispidus*) is the most used animal species for studying infectious diseases due to its unique susceptibility to human pathogens (26). To prepare antibodies for *in vivo* studies using the cotton rat RSV infectious model, we generated camelid single-domain antibodies by immunizing llamas with a recombinant cotton rat pIgR protein (cpIgR) containing five ectodomains (D1-5). Antigen specific B-cells were isolated from llama PBMCs using fluorescence activated cell sorting (FACS) and then V_H_H candidates with confirmed binding to cpIgR by ELISA were sequenced (protocols described in Methods). Forty-five unique V_H_H sequences were selected and recombinantly expressed as fusions to the human IgG1 Fc domain (V_H_H-hFc). The cell binding of recombinant anti-pIgR V_H_H-hFc constructs was validated by flow cytometry using MDCK cells that had been engineered to overexpress cpIgR, human pIgR (hpIgR) or mouse pIgR (mpIgR). All forty-five molecules showed binding to cpIgR-MDCK cells, with thirty-seven cross-reactive to all three species (**Supplementary Table S1**) and no binding to the parental MDCK cells. Surface plasmon resonance (SPR) affinities were measured using BIAcore and indicated that all anti-pIgR V_H_H-hFc molecules bound to recombinant cpIgR (D1-5) with K_D_ values ranging from 16 pM to 200 nM (**Supplementary Table S1**). All thirty-seven molecules having cross-reactivity to three species in the cell binding analysis also exhibited binding to recombinant ECD proteins of hpIgR and mpIgR in the SPR affinity analysis. Epitope binning analysis was conducted against cpIgR ECD protein (D1-5) using a high-throughput SPR system and six non-overlapping bins were identified (**Supplementary Table S1**). To test the domain-level epitope binding of anti-pIgR V_H_H-hFc molecules, we generated recombinant cpIgR domain constructs (denoted D1, D1-2, D1-3, and D4-5), each encoding one or multiple pIgR ectodomain fragments (**Supplementary Table S2**). Results of the ectodomain binding analysis are shown in the checkerboard diagram of **Figure 1B** as well as in **Supplementary Table S1** along with the binning data for tested antibodies. In brief, thirty-six molecules, binding to cpIgR domain constructs of D1, D1–2 and D1-3, were identified as domain-1 (D1) binders and all of them recognized the same epitope region denoted as bin 1 in **Supplementary Table S1**. Three clones, binding to cpIgR D1–2 and D1–3 constructs, displayed as domain-2 (D2) binders with two epitope bins (bin 2 and bin 3). Two clones binding to cpIgR D1–3 construct alone displayed as domain-3 (D3) binders with two epitope bins (bin 4 and bin 6), and two clones binding to cpIgR D4–5 construct displayed as domain-4,5 (D4-5) binders with one epitope (bin 5).

### Transcytosis of anti-pIgR V_H_H-hFc molecules in pIgR-MDCK cells

To evaluate the transcytosis activity of anti-pIgR V_H_H-hFc molecules, we performed a transcytosis assay (**Figure 1A**) using monolayers of cpIgR-MDCK cells grown on Transwell inserts (27). Paracellular leakage was analyzed using Dextran Texas red dye and was found to be minimized after 3 days of culture. For screening purposes, 100 nM of anti-pIgR V_H_H-hFc or control molecules were applied to the basolateral side of polarized MDCK cells at 37 °C. The concentrations of transcytosed antibodies present in the supernatants at the apical side were quantified at 24 hours. Forty-three anti-pIgR V_H_H-hFc molecules were effectively transported across the cpIgR-MDCK cell monolayer, with apical concentrations ranging from approximately 0.9 to 2.2 nM. Two molecules, VHH1 and VHH34, demonstrated high transcytosis activity comparable to that of dIgA, the natural ligand of pIgR (**Figure 1B**, **Supplementary Table S1**). A minimal level of transcytosed antibody was detected for isotype V_H_H-hFc control. Parental MDCK cells that were not transfected with cpIgR did not exhibit transcytosis of any of the antibodies (data not shown). To assess whether the transcytosis activity of the pIgR antibodies is associated with the target domain, the anti-pIgR V_H_H-hFc molecules were grouped by domain mapping and plotted against their quantified transcytosis activity (**Figure 1B**). The similarity of transcytosis activity among the different domain binder groups indicated no strong correlation between binding domains and transcytosis at 24 hours post-treatment. It suggests effective transcytosis of anti-pIgR antibodies can be achieved through targeting multiple pIgR ectodomains.

### Relationship between binding kinetics and transcytosis activity of anti-pIgR V_H_H-hFc molecules

**Figure 2:**
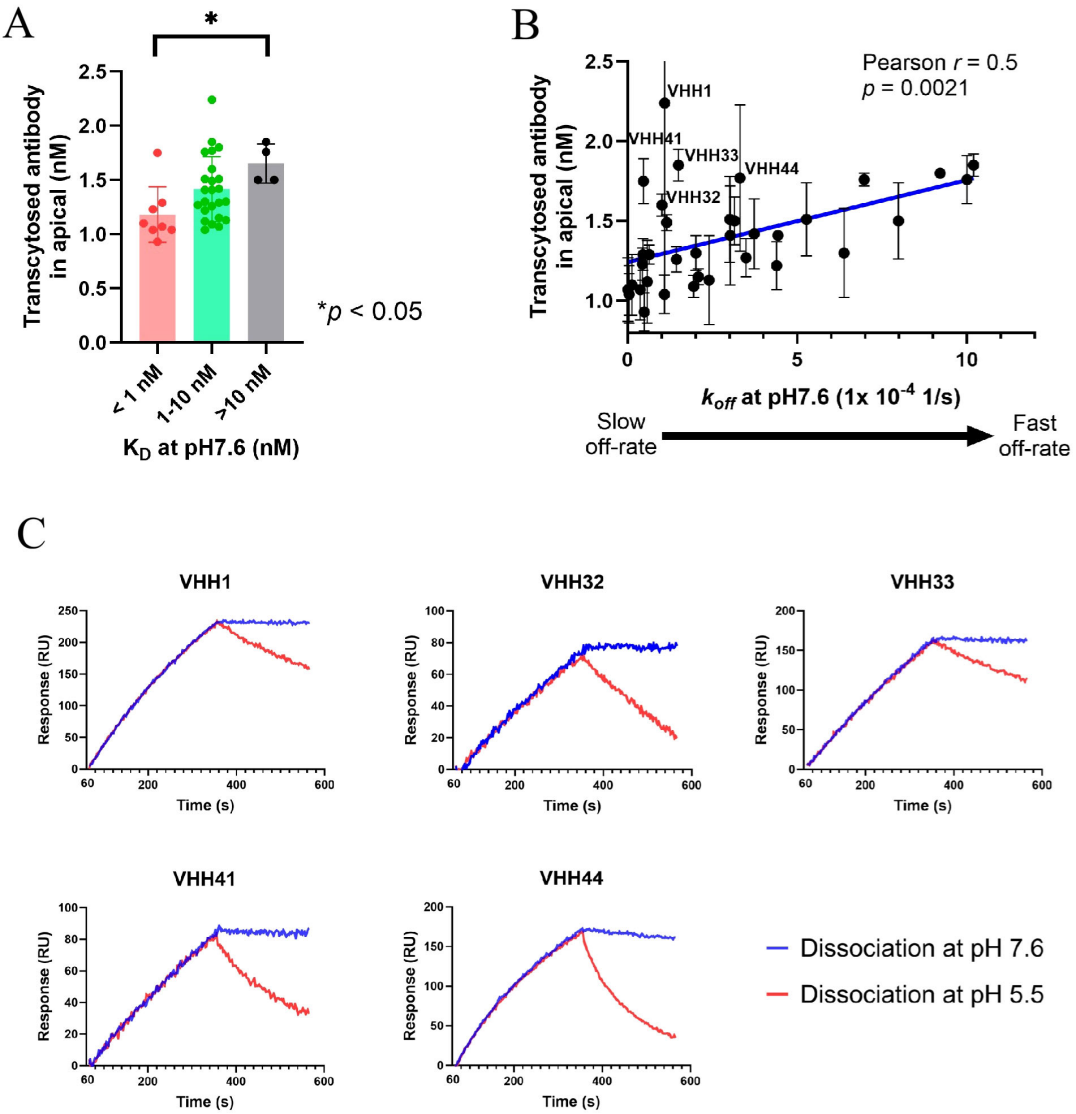
Transcytosis activity and binding kinetics of anti-pIgR V_H_H-hFc molecules having the same epitope on cpIgR. **(A)** Comparison of transcytosis activities among V_H_H-hFc molecules grouped by affinity into three K_D_ ranges: < 1 nM, 1–10 nM and > 10 nM. Each dot represents the average transcytosis of three replicates for one antibody. Standard deviation bars for each affinity group are shown in the graph. Significance measurement was calculated by Ordinary one-way ANOVA analysis, with "*" indicating *p* < 0.05. **(B)** The scatter plot illustrates a positive linear relationship between dissociation rates (*k_off_*) at pH7.6 (x-axis) and transcytosis activity (y-axis) for 36 V_H_H-hFc molecules. The best fit line generated from a simple linear regression analysis is shown in blue. Each data point represents as mean ± SD of three replicates. The Pearson Correlation coefficient (*r*) and significance were calculated and indicated in the graph. Five V_H_H-hFc molecules with high transcytosis and affinities smaller than 5 nM are highlighted with their clone ID in the graph. An arrow diagram illustrates the dissociation kinetics transitioning from slow to fast rates, corresponding to an increasing *k_off_* value along the x-axis. **(C)** Surface plasmon resonance (SPR) analysis was performed to test the binding of five V_H_H-hFc molecules [highlighted in the graph **(B)**] to cpIgR ECD with the association at pH 7.6 following the dissociation at pH 7.6 (blue) vs. pH 5.5 (red). Antibodies were captured on chips and then exposed to 100 nM cpIgR protein for 300s followed by a dissociation step at different pH. Antibody clone ID is indicated in each SPR graph. Graphs represent magnitude of response (RU) over time.

Previously, it has been shown that endosomal sorting during transcytosis of transferrin receptor (TfR) antibodies in endothelial cells can be affinity-dependent, with transcytosis efficiency inversely correlated to the proportion of internalized antibody being sorted into late endosomes and lysosomes (28). For the pIgR antibodies, few studies have explored the relationship between binding affinity and transcytosis activity across epithelial cells. Hence, we utilized the panel of V_H_H-hFc molecules targeting the same epitope bin on cpIgR, spanning a wide affinity range, to investigate whether transcytosis capacity is influenced by affinity or other binding properties. To test this, we categorized the anti-pIgR V_H_H-hFc molecules into three groups based on their affinities: sub-nanomolar (< 1 nM), intermediate (1–10 nM) and low affinity (> 10 nM). As shown in **Figure 2A**, binders with K_D_ values greater than 10 nM exhibited approximately 1.5-fold higher concentration of transcytosed antibodies on the apical side compared to those with K_D_ values below 1 nanomolar (*p* < 0.05). Similar to observations with anti-TfR antibodies, tight binding of anti-pIgR V_H_H-hFc molecules might promote sorting of internalized antibodies toward late endosomes and lysosomes, thereby resulting in a low level of transcytosis. We further examined the relationship between transcytosis capacity and binding kinetics of the anti-pIgR V_H_H-hFc molecules. Plotting the dissociation rates (*k_off_*) against *in vitro* transcytosis activity revealed a positive correlation (Pearson *r =* 0.5, *p* = 0.0021), indicating that poor transporters displayed slower *k_off_* rates compared to potent transporters (**Figure 2B**). However, association rates (*k_on_*) were similar across all tested molecules and showed no significant correlation with transcytosis activity (**Supplementary Figure S1**). These findings suggest that dissociation rate may be a key kinetic parameter influencing the transcytosis activity of this group of anti-pIgR V_H_H-hFc molecules targeting pIgR D1 domain.

In the correlation analysis between transcytosis capacity and binding affinity or kinetics, we observed that several clones (VHH1, VHH32, VHH33, VHH41, VHH44) exhibited significantly high transcytosis levels despite having K_D_ values smaller than ~5 nM and slow off-rates (these clones are highlighted in **Figure 2B**). Previous studies have shown that pIgR-IgA endosomes along the transcytotic pathway in polarized MDCK cells had an acidic pH prior to delivery to the apical compartment (29). If anti-pIgR V_H_H-hFc molecules internalized into cells facilitate the transcytotic pathway similarly to dIgA, we asked whether reduced affinity at acidic pH might influence endosomal sorting and increase transcytosis activity for these five anti-pIgR antibodies. To test this, we performed pH-dependent binding analysis using BIAcore. All five potent transporters demonstrated significantly decreased affinities at pH 5.5 (representing late endosome pH) as compared to pH 7.6. This decrease was primarily driven by approximately 10- to 20-fold increase in dissociation rates (**Table 1**). The pH-sensitive binding of those clones was further confirmed by comparing their dissociation rates at pH 5.5 and pH 7.6 following association at pH 7.6 (**Figure 2C**), mimicking the endosomal pH conditions exposed to pIgR-bound antibodies before apical transport. In addition, we evaluated other clones for binding to cpIgR at both pH 7.6 and pH 5.5. Of the 19 clones with affinities comparable to the five potent transporters (K_D_ between 1 and 10 nM), 12 showed <10-fold increase in dissociation rate at pH 5.5 versus pH 7.6, indicating predominantly pH-independent binding among the clones with relatively low transcytosis (**Supplementary Table S3**). However, pH-sensitive binding appeared to have limited impact on transcytosis activity for strong binders with sub-nanomolar K_D_ values as well as binders with K_D_ values greater than 10 nM (data not shown). The results suggest that pH-dependent binding may enhance transcytosis for some anti-pIgR antibodies whereas it is not a universal characteristic. Other, yet unidentified antibody properties likely also contribute to the regulation of the transcytosis pathway of pIgR-bound antibodies.

**Table 1:** Binding kinetics at pH7.6 vs. pH 5.5 for five high-efficiency transporter V_H_H-hFc molecules that display pH-sensitive binding to cpIgR ECD.

Sample clone ID	pH 7.6			pH 5.5			Ratios (pH5.5/pH7.6)		
	k*_on_* (1/Ms)	k*_off_* (1/s)	K_D_ (M)	k*_on_* (1/Ms)	k*_off_* (1/s)	K_D_ (M)	K_D_ ratio	k*_off_* ratio	k*_on_* ratio
VHH1	5.1E+04	1.1E-04	2.2E-09	4.0E+04	1.5E-03	3.8E-08	17.6	14.0	0.8
VHH32	4.3E+04	1.0E-04	2.4E-09	7.7E+04	8.3E-04	1.1E-08	4.6	8.2	1.8
VHH33	3.5E+04	1.5E-04	4.3E-09	6.1E+04	2.2E-03	3.5E-08	8.2	14.4	1.7
VHH41	6.5E+04	4.7E-05	7.2E-10	8.4E+04	1.1E-03	1.3E-08	18.2	23.2	1.3
VHH44	6.5E+04	3.3E-04	5.1E-09	1.6E+05	3.1E-03	2.0E-08	3.9	9.4	2.4

### Design, generation and characterization of bi-specific antibodies targeting RSV and pIgR

**Figure 3:**
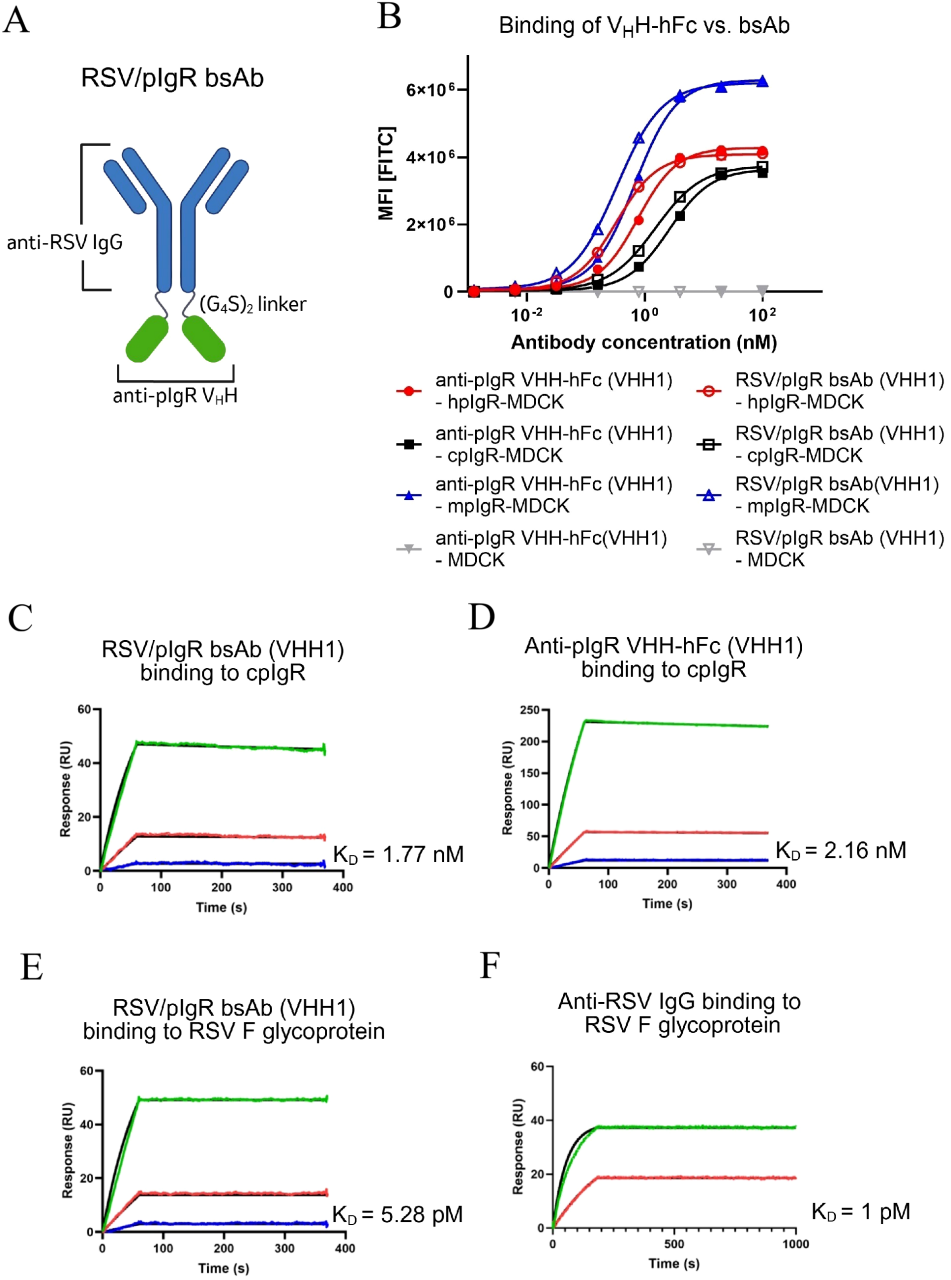
Binding characterization of bsAb construct targeting pIgR and RSV. **(A)** Schematic representation of the RSV/pIgR bsAb construct. Created in BioRender. Liu, J. (2025) https://BioRender.com/z4fba3r. **(B)** Flow cytometry analysis for the binding to MDCK cells stably expressing human pIgR (hpIgR), cotton rat pIgR (cpIgR) and mouse pIgR (mpIgR). Antibodies as indicated in the graph. **(C–F)** SPR analysis for the binding kinetics of antibodies to the full ECD of cpIgR **(C, D)** and RSV F glycoprotein **(E, F)**. Antibody concentrations examined in each SPR analysis included 200 nM (green), 40 nM (red), 8 nM (blue) and 0 nM (not included in graph), except for the analysis of anti-RSV IgG binding to RSV F protein which was tested at 200 nM and 40 nM. Association and dissociation are displayed along with fitted curves (black). The association and dissociation were measured for 60s and 300s as shown in the graphs, except for anti-RSV IgG binding to RSV F protein, where the association and dissociation were extended to 200s and 800s, respectively. Antibodies, antigens and K_D_ values are indicated in the graphs. Graphs represent magnitude of response (RU) over time.

We aimed to develop a dual-functional bsAb that could both neutralize RSV with high potency and efficiently undergo transcytosis across mucosal barriers. For effective RSV neutralization, we selected an anti-RSV IgG that is highly selective to the RSV F glycoprotein and has been previously developed as a potent RSV inhibitor using a standard antibody format (details shown in Methods). Recent studies have reported that clustering of cell surface receptors through bivalent binding of antibodies augments the rate of endocytosis (23, 30). Hence, we generated a bsAb by fusing anti-pIgR V_H_H domains to the C-terminus of the anti-RSV IgG Fc to achieve a 2 x 2 format with bivalent binding for each target, referred to as IgG-V2 (**Figure 3A**). The IgG Fc and V_H_H domains were linked by a (G_4_S)_2_ linker in the bispecific construct. In addition, the full-length IgG format contains a fully functional Fc region that can mediate effector functions and extend serum half-life. To make the bsAb, the top pIgR binding clone, VHH1, was selected using the following rationale (1): cross-reactive to human, mouse and cotton rat pIgR; (2) highly efficient *in vitro* transcytosis which was comparable to dIgA in the MDCK monolayer-based transcytosis assay; (3) high yield and optimal purity expression and production as a V_H_H-hFc construct (data not shown). The RSV/pIgR bsAb construct was expressed using the ExpiCHO expression system and purified with high yields and high homogeneity of the major species representing the full bsAb molecule (**Supplementary Table S4-1**). Mass spectrometric analysis of purified bsAb did not detect significant protease cleavage at the linker region between RSV IgG and V_H_H insert (**Supplementary Table S4-2**).

To assess whether fusing anti−pIgR V_H_Hs to the C−terminus of the RSV antibody affected target recognition, we measured bsAb binding to cpIgR, hpIgR and mpIgR expressed on MDCK cells by flow cytometry. The bsAb retained strong cell binding comparable to the parental anti-pIgR V_H_H−hFc, with EC_50_ values of 1.53 nM (cpIgR), 0.33 nM (hpIgR) and 0.34 nM (mpIgR) (**Figure 3B**). BIAcore analysis confirmed comparable binding affinities of the bsAb and parental V_H_H−hFc to the full ECD of cpIgR (**Figures 3C, D**). Additionally, binding of the bsAb to the RSV F protein was confirmed by BIAcore in comparison to the parental anti-RSV IgG without the V_H_H fusion. Both bsAb and anti-RSV IgG exhibited very strong affinities, with K_D_ values in the single-digit picomolar range (**Figures 3E, F**).

### Transcytosis and RSV neutralization functions of RSV/pIgR bsAb

**Figure 4:**
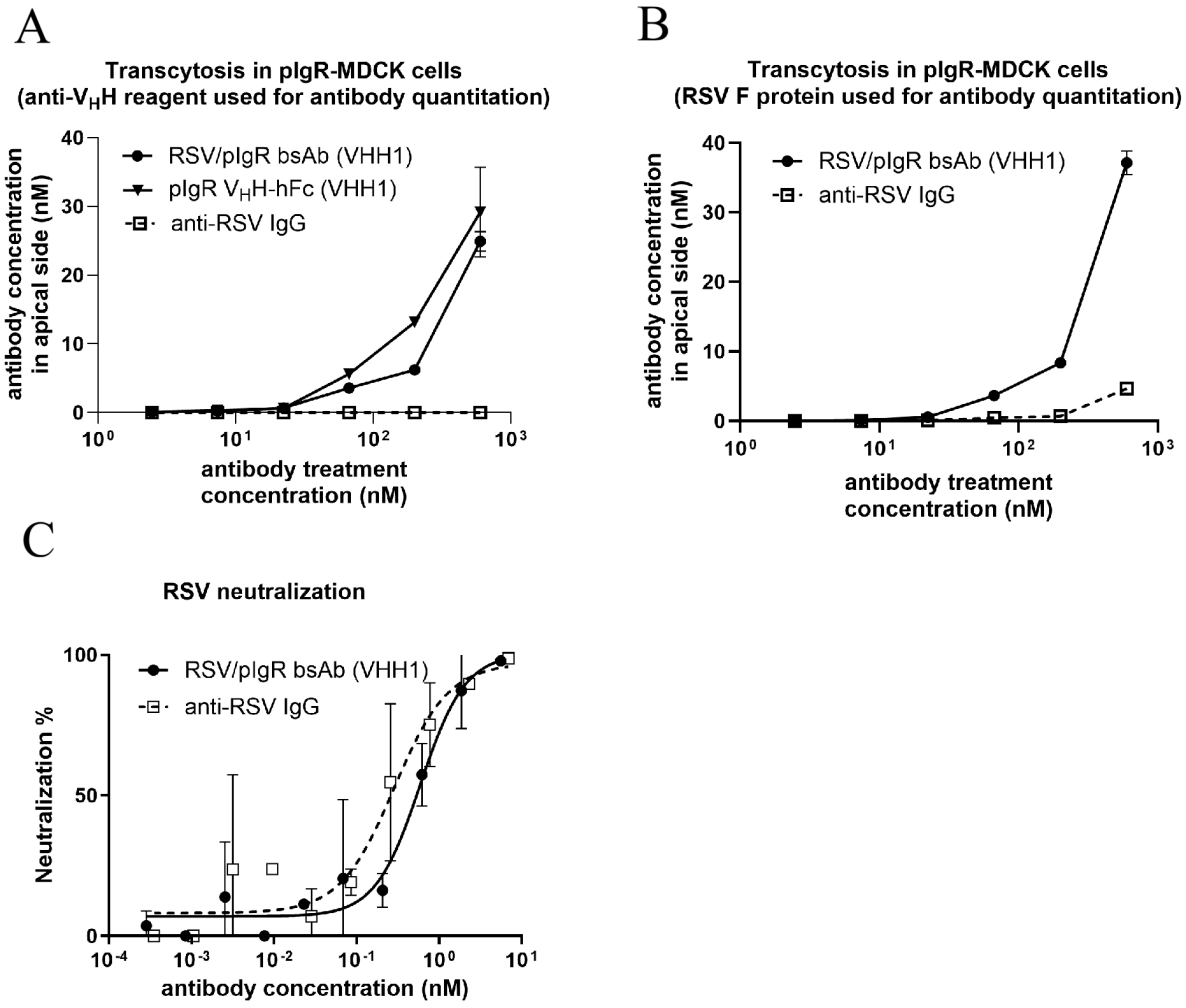
Functional characterization of RSV/pIgR bsAb. **(A, B)** Transcytosis activity of antibodies in cpIgR-MDCK cells at 24 hours after treatment with a series of antibody dilution starting from 600 nM. Transcytosed antibodies were quantified using electrochemiluminescence method with immobilized anti-V_H_H protein **(A)** or RSV F protein **(B)** as the capture reagent on working electrode. **(C)** RSV neutralization function of bsAb compared to parental anti-RSV IgG. Viral neutralization percentage relative to controls without antibody treatment is plotted vs. antibody treatment concentration. Values are presented as mean ± SD of three replicates.

To evaluate whether the RSV/pIgR bsAb maintained the transcytosis function of the anti-pIgR V_H_H formatted as a C-terminal fusion in the bsAb construct, we assessed the transcytosis activity of both the bsAb and the parental anti-pIgR V_H_H-hFc molecule in cpIgR-MDCK cells, using a series of antibody concentrations starting at 600 nM. At 24 hours post-treatment, the apical media containing transcytosed antibodies was collected and split into two aliquots. One aliquot underwent the quantification of transcytosed antibody using an electrochemiluminescence-based method with anti-camelid V_H_H antibody as a capture reagent. The data demonstrated the anti-pIgR V_H_H moiety fused in the bsAb was able to facilitate transcytosis at a similar level as the parental V_H_H-hFc molecule (**Figure 4A**). The other aliquot of apical media was also examined using the electrochemiluminescence-based quantitation but with RSV F protein as the capture reagent that can bind to the anti-RSV IgG. The quantified concentrations of transcytosed antibodies against RSV F protein were consistent with the results measured using anti-V_H_H capture reagent (**Figure 4B**). These results indicate that the anti-pIgR V_H_H can effectively transport IgG as a cargo without abolishing the IgG target binding capability.

We examined if the bsAb construct retained the anti-RSV IgG potency in viral neutralization. Using the RSV neutralization assay described in the Methods, we showed that the bsAb construct featuring the anti-RSV IgG displayed highly potent neutralization activity that was comparable to the parental anti-RSV IgG, with IC_50_ at 0.5 nM (**Figure 4C**). The result indicates that the addition of the anti-pIgR V_H_H moiety did not significantly alter the potency of anti-RSV IgG in bsAb format.

### Transcytosis activity of RSV/pIgR bsAb in human primary ALI model and its serum clearance *in vivo*

**Figure 5:**
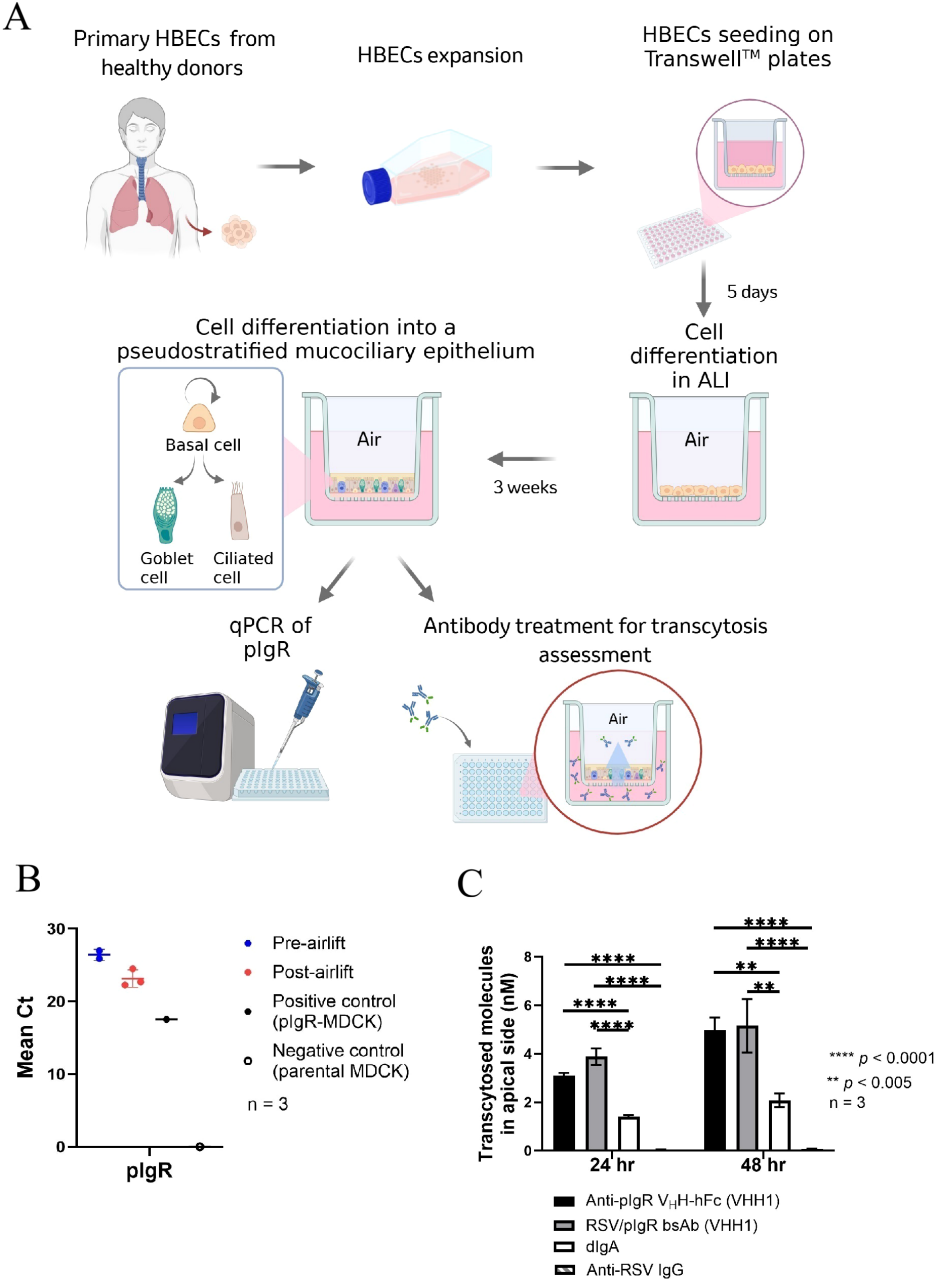
Transcytosis of RSV/pIgR bsAb in human lung air-liquid interface (ALI) model. **(A)** Schematic diagram of the ALI cultures of human bronchial epithelial cells (HBECs). Created in BioRender. Liu, (J) (2025) https://BioRender.com/usxo31l. **(B)** qPCR for measuring the pIgR mRNA expression in cells collected from the ALI model at different stages of cell differentiation, pre-airlifting and post-airlifting. Cultures derived from three healthy donors were tested. MDCK cells expressing human pIgR and parental MDCK cells were used as positive and negative controls, respectively. **(C)** Transcytosis activity of anti-pIgR V_H_H-hFc, RSV/pIgR bsAb, anti-RSV IgG and dIgA in the lung ALI model. Each sample was treated basolaterally at 600 nM. After 24 hours or 48 hours post-treatment, 100 µL of fresh media was added to the apical side to hydrate the mucus for 30 minutes and then collected for quantifying the levels of transcytosed antibodies. Transcytosed antibody concentrations in apical media are plotted per construct (n = 3). Error bars represent mean ± SEM in the graph. The anti−RSV IgG control shows minimal transport and appears as a barely visible bar. Significance measurements were calculated by Ordinary one-way ANOVA for each 24 h or 48 h treatment period. "**" and "****" indicate *p* < 0.005 and *p* < 0.0001, respectively.

To determine if the anti-pIgR V_H_H shuttle can direct the antibody distribution into the human lung lumen, we employed an air-liquid interface (ALI) model using primary human airway bronchial epithelial cells (HBECs) to assess the transcytosis activity of RSV/pIgR bsAb containing the VHH1 clone. For the ALI model, primary human bronchial epithelial cells collected from healthy donors were cultured and differentiated to establish an *in vitro* culture system that closely mimics *in vivo* structure and function (**Figure 5A**) (31, 32). The mRNA level of pIgR in cells of the ALI model was assessed by qPCR at both pre- and post-airlifting stages and demonstrated a stable expression of hpIgR in both primary and differentiated cells (**Figure 5B**). Once the ALI model was established, cells were treated basolaterally with 600 nM of antibodies, and apical mucus was collected at 24 and 48 hours to assess transcytosis. Both the monospecific anti-pIgR V_H_H-hFc and the RSV/pIgR bsAb were tested alongside dIgA and the parental anti-RSV IgG. As expected, the RSV/pIgR bsAb and anti-pIgR V_H_H-hFc constructs exhibited markedly higher apical transport than the parental anti-RSV IgG which does not contain the anti-pIgR moiety (**Figure 5C**). Surprisingly, both V_H_H-hFc and bsAb showed almost a two-fold higher concentration in the apical mucus than that of the dIgA, despite the anti-pIgR V_H_H-hFc molecule displaying slightly lower transcytosis activity than dIgA in the *in vitro* transcytosis assay. The inconsistency might be attributed to the different experimental conditions between the two models, including distinct cell types, differential pIgR expression level and different antibody treatment concentrations. Overall, our findings suggest that the dual-functional bsAb of an IgG-V2 (2 x 2) format with bivalent pIgR V_H_H domain fusions can be an effective modality for mucosal enrichment of large molecules like antibodies or antibody-based therapeutics.

To examine the RSV/pIgR bsAb *in vivo*, we evaluated the pharmacokinetic (PK) profile in cotton rats. The bsAb and the original anti-RSV IgG were administered intramuscularly in healthy cotton rats, followed by serum collection at 0, 1, 6, 24, 48, 96 and 240 hours. The RSV/pIgR bsAb displayed very low serum levels, less than 1 µg/mL, through all testing time points, whereas the parental anti-RSV IgG showed stably increasing serum levels from ~10 µg/mL at 1 hour to ~50 µg/mL at 24–48 hours (**Supplementary Figure S2A**). The normal half-life of the anti-RSV IgG control suggested that the accelerated clearance observed with bsAb is likely driven by attributes of the C-terminal V_H_H Fc fusion rather than a systemic issue from models or assay. Polyspecific binding of the anti-pIgR V_H_H-hFc molecule (VHH1) was evaluated by polyspecific reagent (PSR) binding assay and found to be minimal (data not shown), excluding non-target binding as a potential contributor to the rapid clearance observed. The low serum concentration of bsAb in cotton rats is more likely due to rapid serum clearance resulting from pIgR-mediated transcytosis in mucosal tissues, and its poor absorption via the intramuscular route may be considered. In addition, we used the intravenous route in wild-type CD1 mice to evaluate PK profiles for three cotton rat/mouse cross-reactive anti-pIgR V_H_H-hFc molecules (VHH1, VHH17 and VHH34). Similar to the PK of the bsAb in cotton rat, the three V_H_H-Fc molecules exhibited rapid serum clearance, with serum concentrations decreasing from 20 - 30 µg/mL to below 1 µg/mL within 24 hours (**Supplementary Figure S2B**). Our PK findings are consistent with previous reports that indicated anti-pIgR V_H_H molecules in either monovalent or bivalent format displayed rapid serum clearance in mice (25). In our mouse PK analysis, VHH1 showed a clearance rate nearly twice as high as VHH17, while VHH17 has approximately 70-fold lower affinity. It suggests affinity dependent exposure for the pIgR antibodies. VHH34, a D4–5 binder with medium affinity, displayed slightly higher serum level than the other two molecules targeting to domain D1 and D3 (**Supplementary Figure S2B**, **Supplementary Table S5**). These findings indicate that both affinity and epitope specificity may influence PK profile and mucosal targeting of anti-pIgR shuttles. Further studies using more physiologically relevant models are needed to fully understand these effects.

## Discussion

Passive immunization with neutralizing RSV antibodies remains the primary prophylactic strategy for infants and young children. Among the five antibody classes, IgG is preferred for therapeutics and prophylactics due to its stability, extended half-life, and ability to enhance immune responses (13). However, unlike dIgA or IgM, IgG lacks affinity for the pIgR on mucosal epithelial cells. Under normal conditions, IgG levels at mucosal surfaces are relatively low compared to systemic circulation. Therefore, enhancing IgG translocation across the epithelium into mucosal compartments is highly desirable for advancing IgG-based therapeutics targeting infectious diseases. Our study aimed to develop single-domain (V_H_H) antibodies capable of active transport across the mucosal barrier via targeting pIgR, serving as shuttle moieties in antibody-based therapeutics. We generated anti-pIgR V_H_Hs and engineered the lead clone into an anti-RSV IgG to form a bsAb that exhibited efficient transcytosis activity in mucosal epithelial cells and primary human airway tissue models, while maintaining full RSV neutralization efficacy. The study highlights the integration of antibody specificity with functionality to achieve preferential distribution to mucosal spaces.

The V_H_H scaffold, small (12-15kDa) and stable, offers many favorable properties as fusion partners for making biologic drugs, including high solubility and maintenance of full antigen-binding capabilities (33). Its robust biophysical stability facilitates straightforward engineering onto IgG scaffolds through flexible GS linkers to form high-quality bsAbs with minimal aggregation or impurities (34). Therefore, we chose V_H_H as the antibody format for developing a mucosal drug delivery platform. Leveraging llama immunization and next-generation sequencing, we discovered forty-five anti-pIgR V_H_H clones with nanomolar to picomolar affinities, targeting six distinct epitope bins predominantly within the D1 domain of cpIgR. Functional screening revealed robust transcytosis activity across cpIgR-MDCK cell monolayers, with similar efficiency across antibodies binding different pIgR ectodomains.

Human pIgR comprises five tandem Ig-like ectodomains (D1-D5) arranged as a compact triangle with an interface between ligand-binding domains D1 and D5 (7). D1 and D5 are the two main domains that bind on the Fc subunits and J-chain of dIgA and subsequently stabilize the pIgR-IgA complex to initiate transcytosis across the epithelium. Up to 50% of unbound pIgR in human also traffics to the apical surface and the full ectodomain (D1-5) is released as an unliganded form (35). This phenomenon may explain why the transcytosis capability of anti-pIgR antibodies is not limited by their binding domain. Beyond the role in mucosal delivery, pIgR domains have other therapeutic relevance. For example, a synthetic peptide representing residues of human pIgR domain 4 was found to inhibit *Streptococcus pneumoniae* adherence to epithelial cells (36). Structural data implicate domains D3 and D4 in bacterial entry (5), highlighting novel intervention points. To our knowledge, our study is the first to report antibodies binding to the D3 domain of pIgR.

By leveraging a set of anti-pIgR V_H_Hs variants targeting the same epitope bin on the D1 domain of cpIgR, our binding affinity analyses revealed a positive correlation between binding K_D_ values and transcytosis activity, with antibodies exhibiting relatively low affinity (K_D_ >10 nM) transcytosing 1.5-fold more efficiently than high-affinity binders (K_D_ < 1 nM). This affinity-dependent transcytosis was mainly driven by differences in dissociation rates. A previous study reported that bivalent constructs of anti-pIgR V_H_Hs enhance hpIgR endocytosis via receptor clustering but do not necessarily improve transcytosis, suggesting additional factors influence pIgR antibody transcytosis beyond internalization (23). Depending on the way antibodies interact with pIgR and the receptors are clustered, bivalent constructs of pIgR antibodies may facilitate the internalization of receptors and antibodies into early endosomes but also direct them toward distinct intracellular fates: (1) sorting to apical recycling endosomes for transcytosis trafficking, (2) recycling back to the basolateral plasma membrane, or (3) being trapped inside cells followed by lysosomal degradation. The latter two trafficking pathways can limit receptor and antibody transport to the apical surface and consequently reduce the transcytosis efficiency. Previous studies of anti-TfR antibodies indicated that reducing affinity could increase total brain accumulation by promoting antibody dissociation from TfR and release into the brain parenchyma (37). Low-affinity binding to TfR redirects antibody transcellular trafficking away from lysosomal degradation and toward enhanced exocytosis at the abluminal side of the blood-brain barrier (28). Our data suggest that dissociation kinetics of anti-pIgR V_H_H-Fc molecules may be a critical parameter for optimizing pIgR-mediated transcytosis.

Previous studies have shown that endosomal acidification facilitates receptor-ligand dissociation required for efficient receptor recycling (38). Hence, we explored pH-dependent binding as a potential attribute influencing intracellular trafficking for transcytosis of anti-pIgR antibodies. Among the anti-pIgR V_H_H variants targeting the same epitope on pIgR D1 domain, potent transporters with K_D_ values between 1 and 5 nM (VHH1, VHH32, VHH33, VHH41, VHH44) demonstrated reduced affinities accompanied with significantly faster dissociation rates at acidic endosomal pH compared to neutral pH. The result agrees with reports that enhanced transcytosis observed for pH-sensitive anti-TfR antibody (39). The pH sensitivity likely enables antibodies to dissociate from pIgR during endosomal acidification, favoring transcytotic routing over degradation. Therefore, enhancing anti-pIgR antibody transcytosis can be achieved through the combined effects of pH-sensitive binding and receptor clustering mediated by bivalent or biparatopic constructs. However, our observations are limited to a specific subset of binders, and pH-sensitive binding may not be a universal feature of all anti-pIgR antibodies. Further studies involving precise molecular characterization of intracellular and endosomal compartments for anti-pIgR antibodies will be valuable to elucidate antibody attributes and intrinsic cellular factors that promote preferential routing into the transcytosis pathway while avoiding degradation.

Targeting of pIgR by chimeric peptides or antibodies has been recognized as an effective way to deliver large molecules and plasmid DNA into mucosal tissues (22, 40–43). To address COVID-19 infection, White et al. generated bifunctional molecules by combining anti-pIgR V_H_H domains and the ACE2 extracellular domain specifically targeting the SARS-CoV-2 spike protein (25). Our study described another therapeutic design, bsAb of an IgG-V2 (2 x 2) format featuring C-terminal pIgR-binding V_H_H domain fused to an anti-RSV IgG. The bsAb facilitated the dual functions of efficient transcytosis and potent RSV neutralization, with preserved antigen binding post-transcytosis. Although a 2 x 1 format (bivalent anti-RSV Fab with a monovalent pIgR-binding V_H_H) could increase dissociation from pIgR, reduced avidity may weaken cell surface capture and limit internalization (23). In contrast, we selected bsAb of an IgG-V2 (2 x 2) format with pH-sensitive V_H_Hs that may balance high avidity for efficient uptake at the cell membrane with rapid dissociation in acidic endosomes, thereby promoting transcytosis and apical release without sacrificing neutralization. Consistent with our concept, Kuhn et al. independently conducted parallel research and developed a bivalent anti-TfR antibody with high affinity and pH-sensitive binding that enables efficient transcytosis to enhance blood-brain barrier delivery (44). The 2 x 2 format also demonstrated favorable production and stability characteristics (34). The comparison of 2 x 2 versus 2 x 1 constructs can be pursued in future work to further optimize transcytosis *in vitro* and *in vivo*. Moreover, we did not employ tandem V_H_Hs at the Fab N-terminus due to considerations on potential steric interference with RSV F protein engagement and neutralization potency. We also considered that V_H_H as a C−terminal fusion on the Fc might interfere with Fc effector function (e.g., reduced FcγRs engagement and antibody-dependent cellular cytotoxicity (ADCC)/antibody-dependent cellular phagocytosis (ADCP)) due to steric and conformational effects (45, 46). Evidence for a protective or deleterious role of ADCC in RSV animal models is limited, and the overall contribution of Fc-mediated effector functions in RSV disease remains uncertain (47). Even if such an effect diminishes cytotoxic activity against infected cells, the neutralizing potency of our anti-RSV IgG was not affected and may still provide sufficient viral neutralization to achieve clinical response. Future RSV antibody therapeutics should be systematically assessed for Fc-mediated effects and characterized to identify safe and effective antibody features.

However, both previous studies and our findings demonstrated a rapid serum clearance of anti-pIgR molecules in healthy rodents, suggesting a target-mediated drug disposition (TMDD) effect. Consistent with these PK profiles of anti-pIgR molecules, recombinant IgA oligomers clear faster than IgG1 and show preferential biodistribution and degradation in liver and small intestine in mice, reflecting active pIgR-mediated handling (12). Previous studies have demonstrated that mice and other types of rodents exhibit high pIgR expression in the liver (48), a characteristic distinct from humans and primates (49). These interspecies differences underscore the importance of species appropriate pIgR profiling and biodistribution analyses when interpreting clearance and designing anti-pIgR therapeutics. This may raise concerns regarding the direct translatability of rodent *in vivo* data to non-human primate (NHP) models or humans. Besides TMDD due to high tissue pIgR burden, another possible contributor to the fast clearance in rodents may be low affinity off-target/polyspecific interactions leading to peripheral retention and catabolism. Thus, our lead anti-pIgR V_H_H-hFc (VHH1) was assessed by PSR binding assay and showed minimal polyspecific binding, making non-target interactions unlikely to be a primary contributor to the observed. Moreover, approaches to extend the half-life of pIgR shuttles, such as optimization of anti-pIgR V_H_H domains and FcRn-mediated half-life extension, warrant exploration. We may refine RSV/pIgR bsAb engineering to improve targeted tissue specificity and transcytosis efficiency. Meanwhile, pharmacokinetics and biodistribution in NHP are needed to establish *in vivo* exposure, mucosal enrichment, and safety profiles in a model with greater translational relevance. Modalities may also be assessed for antiviral activity and mucosal delivery in human airway epithelial ALI cultures infected with RSV, enabling direct measurement of apical transport and viral neutralization in a physiologically relevant model. These studies will collectively inform the clinical development path and facilitate comparison with current IgG standards of care.

In conclusion, as a mechanism to overcome challenges for protecting infants and young children from RSV infection, we generated a panel of anti-pIgR V_H_H antibodies that can effectively facilitate pIgR-mediated transcytosis across the mucosal barrier and serve as a shuttle moiety fused with anti-RSV IgG for increasing the mucosal exposure of IgG-based biologics. These findings, along with our observation on the affinity-dependent and pH-dependent transcytosis of anti-pIgR antibodies provide a framework to expand antibody-based treatments for respiratory viral infections.

## Materials and methods

### Cell lines and experimental materials

Madin-Darby canine kidney (MDCK) cell line, commonly used for assessing transcytosis activity in epithelial cell models, was obtained from the American Type Culture Collection (ATCC, Manassas, Virginia, Cat. CCL-34) and cultured in Eagle’s Minimum Essential Medium (ATCC, Cat. 30-2003) containing 10% fetal bovine serum (FBS) at 37°C with 5% CO_2_. Using a previously published protocol (23), we developed MDCK cell lines stably expressing full-length hpIgR, mpIgR and cpIgR, respectively.

The generation of human RSV F glycoprotein was described previously (50). Anti-RSV IgG used in our study is a fully human IgG1 monoclonal antibody targeting a region of the RSV fusion protein. It was generated and developed internally, showing potent inhibition on RSV. dIgA protein used in our study was recombinantly produced according to an IgA2m2 production method described previously (12), except for using CaptureSelect™ IgA Affinity Matrix (Thermo Fisher Scientific, Waltham, MA) for affinity chromatography. In brief, RSV variable domains (derived from our internal anti-RSV IgG described above) were combined with the human IgA2m2 heavy chain constant domain variants shown to improve dimer assembly (e.g., I458V, A478V) to be expressed recombinantly. Assembly of the light chain, IgA2m2 heavy chain and J chain yielded IgA2m2 protein pool consisting of monomer, dimer, and polymer as purified by the IgA Affinity Matrix. Then, the dimer peak was purified with HiLoad 16/600 Superdex 200pg column (Cytiva, Marlborough, MA) with AKTA Avant system (Cytiva). The final purity of the dimer by SEC was >97%.

### Construct design, protein expression and protein purification

To advance antibody generation and subsequent characterization, we generated ectodomain 1-5 (D1-5) of human pIgR (1–547 AA), murine pIgR (1–549 AA), and cotton rat pIgR (1–547 AA), along with truncated ectodomain variants of cotton rat pIgR (D1, D1-2, D1-3, and D4-5). The domain boundaries of cotton rat pIgR, shown in **Supplementary Table S2**, were identified by aligning with sub-domain amino acid sequences of human pIgR published previously (7). Gene blocks encoding the desired pIgR sequences with a C-terminal 6x His-tag were synthesized and cloned into mammalian expression vectors (Azenta Life Sciences, Burlington, MA). pIgR proteins were expressed in ExpiCHO™ cells and then purified using immobilized metal ion chromatography (GE healthcare, Chicago, IL) according to the manufacturer’s instructions.

RSV/pIgR bsAb was constructed by fusing anti-pIgR V_H_H to the C-terminus of the anti-RSV IgG heavy chain via a (G_4_S)_2_ linker. Gene blocks encoding the full heavy chain or light chain were synthesized and cloned into customized mammalian expression vector (Azenta Life Sciences). Plasmids encoding bsAb constructs were transfected into ExpiCHO™ cells using ExpiFectamine™ transfection kit (Thermo Fisher Scientific). After 7 days, supernatant was harvested, filtered and purified by Protein A based MabSelect™ SuRe affinity chromatography on an AKTA express system. Eluted fractions were immediately neutralized using 25% (v/v) 2M Tris-HCl pH 7.0, dialyzed to PBS, and stored at 4°C.

Protein characterization was performed using Ultra Performance Size Exclusion Chromatography (UP-SEC) and capillary gel electrophoresis with sodium dodecyl sulfate (CE-SDS). An overall purity of >90% by UP-SEC was used as the acceptable criterion for bsAb and pIgR proteins. Purified bsAb was analyzed by mass spectrometry to confirm molecular weight and structural integrity. Reduced or non−reduced samples (1 µg, at 0.2 µg/µL) were injected onto a PLRP-S column (1000 Å, 5 µm, 50 × 2.1 mm) on an Agilent RPLC system, with the chromatographic effluent coupled directly to an Agilent 6230 time−of−flight (TOF) mass spectrometer for intact mass analysis. The observed mass spectra matched the expected theoretical mass of the bsAb, confirming successful purification and correct assembly.

### Generation and screening of anti-pIgR antibodies

All animal procedures described in this study were approved by the Institutional Animal Care and Use Committee of Capralogics, Inc., a USDA regulated research facility. All animal work performed were in compliance with the Guide for the Care and Use of Laboratory Animals published by the National Research Council (51), the American Veterinary Medical Association Guidelines for the Euthanasia of Animals (52), and the ARRIVE guidelines (53).

To generate a panel of anti-cpIgR single-domain antibodies, we immunized llamas with purified cotton rat pIgR (cpIgR) protein, comprising five ectodomains (D1-D5), via four subcutaneous injections at three-week intervals at Capralogics, Inc. Prior to whole blood collection, high antibody titers in sera collected from immunized llamas were screened against cpIgR protein by ELISA. Peripheral blood mononuclear cells were isolated from whole blood followed by surface staining with using biotinylated cpIgR, goat anti-llama IgG (H+L) FITC conjugate (Thermo, Cat. A16061), 7-AAD live/dead cell stain (Biolegend, Cat. 420404), and streptavidin BV421 (Biolegend, Cat. 405226). Live B-cells, positive for binding to biotinylated antigen and IgG, were sorted as single cells into 96-well plates using a BD FACSAria Fusion. The single sorted cells were cultured for two weeks with gamma irradiated CD40L-EL4 recombinant cells and internally made llama IL-2/IL-21 cytokines at 37°C. After two weeks, B-cell culture supernatants were screened using ELISA and flow cytometry for binding to cpIgR. Candidates of interest were then lysed in Qiagen TCL buffer with 1% B-mercaptoethanol, followed by total RNA extraction using Qiagen Turbocapture protocol (Qiagen, Cat. 72251).

V_H_H sequences for all selected hits were identified using the protocol described previously (54). In brief, cDNA was generated using SuperScript IV reverse transcriptase (Thermo, Cat. 18090050) in the presence of a template switching oligo (TSO) for 5’RACE. Two PCR rounds with GoTaq polymerase (Promega, Cat. M7422) and custom primers amplified the V_H_H region and added Illumina MiSeq adapters and indices for multiplexed NGS. Unique V_H_H sequences that showed binding to pIgR were sent to GeneScript Biotech Corporation (Piscataway, NJ) for small-scale expression and production of recombinant V_H_H-hFc molecules in ExpiCHO cells following the manufacturer’s protocol in a V_H_H-hIgG1 Fc format.

### Surface plasmon resonance

Binding kinetics studies were conducted using BIAcoreTM 4000 surface plasmon resonance (SPR) system (Cytiva). The running buffer was filtered HBS-EP (10 mM Hepes,150 mM NaCl, 3.4 mM EDTA, 0.05% polysorbate 20, pH7.6). To measure binding affinity of pIgR antibodies (V_H_H-hFc molecules and RSV/pIgR bsAb) to pIgR ECD proteins of different species (including cotton rat, human and mouse), purified pIgR antibodies was captured on CM5 chips on which anti-human Fc antibody (Cytiva Cat. 29234600, Lot 10330564) has been immobilized using standard amine coupling chemistry. A concentration series of pIgR protein (0–200 nM, 1:5 dilution) was injected over antibodies and reference surfaces at a flow rate of 30 µL/min. The association and dissociation were measured for 60s and 300s at 25°C. After each sample injection, the surface was regenerated by a 30s injection of 3 M of MgCl_2_. Affinity and kinetic constants were determined by fitting the sensorgrams with the 1:1 Langmuir model using BIAcore 4000 evaluation software (Cytivia). To test the binding of pIgR antibodies to individual ectodomains of cpIgR, captured pIgR antibodies were exposed to serially diluted ectodomain cpIgR proteins (D1, D1-2, D1-3, D4-5). In some cases, RSV F protein replaced pIgR to assess bsAb and anti-RSV IgG binding. Anti-RSV IgG binding to RSV F protein was tested at 200 nM and 40 nM with extended association time and dissociation time of 200s and 800s, respectively. For low pH binding kinetics, the running buffer was replaced with filtered MES buffer (10mM MES, 150mM NaCl, 0.05% (v/v) Tween-20, pH 5.5). This buffer was also employed as the dissociation buffer at pH 5.5 for the pH-dependent off-rate analysis. Epitope binning analysis of anti-pIgR V_H_H-hFc molecules was performed using Carterra’s high-throughput SPR technology platform (LSA instrument, Carterra, Salt Lake City, USA). A classical sandwich assay format was employed to characterize antibody epitopes. Briefly, primary antibodies were diluted to 10 µg/mL and captured on HC30M sensor chips (Carterra) via amine-coupling using the LSA’s surface array preparation protocol. The antibody-coated chips were then sequentially exposed to 100nM cpIgR ECD protein for 180s, followed by a secondary antibody at 50 μg/mL for additional 180s. Raw data from the high-throughput epitope binning were analyzed using the LSA epitope software.

### Flow cytometry

To evaluate the binding specificity and cross-species reactivity of pIgR antibodies, we used MDCK cells that stably express full-length pIgR of cotton rat, human and murine, respectively. Cells were split into equal fractions (50,000 cells/well) and incubated with a series of anti-pIgR antibody concentrations starting at 100 nM for 30 minutes at 4°C, followed by washing 2X with cold blocking buffer (PBST supplemented with 3% FBS) and incubation with a fluorescently labeled anti-human Fc antibody (Jackson Immuno-Research, 109-606-170) at 2 μg/ml for 30minutes. After another 2X washes with cold blocking buffer, cells were resuspended in running buffer (PBS) and analyzed with an iQue Screener (Intellicyt Corporation, Albuquerque, NM). Binding was assessed by RL1 (A647) MFI from the live cell population and EC_50_ was calculated by fitting log concentration of antibodies versus MFI in Prism (GraphPad).

### Polyspecificity reagent assay

The polyspecificity reagent (PSR) binding assay was performed as previously described (55). Cytosolic and membrane protein fractions were prepared and biotinylated using standard protocols. Non-target polyspecific binding of anti-pIgR V_H_H-hFc was assessed with polystyrene beads coated with polyclonal goat anti-human Fc antibody; anti-pIgR V_H_H-hFc was captured on the coated beads, incubated with the biotinylated protein preparations, and detected with a fluorescent streptavidin probe. Polyspecific binding was quantified on an Attune NxT flow cytometer (Thermo Fisher Scientific), and results were reported as fold change in fluorescence relative to background (beads incubated without antibody).

### Transcytosis activity in MDCK cells

We used MDCK cells and engineered MDCK cells over-expressing pIgR to assess the transcytosis activity of tested antibodies. To prepare polarized epithelial monolayers, 3 × 10^4^ cells per well were seeded on Transwell™ permeable supports containing 0.4 μm polycarbonate membrane filter (Corning LLC., Arizona, HTS 96-well plate Cat. 3391) and cultured for 3 days to form monolayers. To ensure the monolayer cells with good integrity, we added Dextran Texas Red (Thermo Fisher Scientific, Cat. L0144-25MG) in the basolateral chamber and incubated for 1 hour. Supernatants from apical chambers were collected to detect if dye contents were leaked through cell monolayer and transwell membrane. Flux range for intact cell monolayer is <2%. After cell leakage test, dye was removed, and cells were washed 3 times with EMEM containing 1% FBS (assay media), starved for overnight prior to treatment. To test the transcytosis activity of pIgR antibodies across cell monolayers, 100 nM of test or control molecules were added to the basolateral chamber and after 24 hours 100 μL of media was collected from the basolateral and apical chambers. The concentrations of tested molecules present in basolateral and apical media was quantified using electrochemiluminescence method by following the manufacture’s protocol (Meso Scale Diagnostics, Rockville, MD). In brief, for the quantification of V_H_H-hFc molecules, we used a biotinylated anti-V_H_H antibody (Genscript, Cat. A01995) as the capture reagent and in-house labeled sulfo-tagged anti-human IgG CH2 domain antibody (Thermo Fisher Scientific, Cat. MA5-16929) as the detection reagent. To quantitate the amount of dIgA present in the basolateral and apical media, we used biotinylated F(ab)2 fragment of anti-human IgA, a chain (Jackson Lab, Cat. 109-066-011) as a capture reagent and sulfo-tagged anti-human IgA, α-chain (Jackson Lab, Cat. 109-005-011) as a detection reagent. The amount of V_H_H-hFc or dIgA in basolateral and apical chambers were calculated by plotting electrochemiluminescence units (ECLU) against corresponding standard curves in Prism (GraphPad).

### Plaque reduction neutralization assay

A plaque reduction neutralization assay has been developed for RSV in house and was performed in this study by following the protocol described previously (56). Briefly, tested antibodies in serial dilutions with OptiMEM medium (Gibco, Cat. 31985–070) were applied into Poly-D-coated 96-well flat bottom plates (Corning Costar, Cat. 354640). RSV strain A Long (ATCC VR-26) diluted at 100 pfu/well were added and mixed with antibodies, followed by incubation for 1 hour at 37 °C with 5% CO_2_. Then, host HEp-2 cells (ATCC CCL-23) at concentration of 1.2 × 10^6^ cells per ml were added to the antibody/virus mixture in each well and incubated for 1 hour. Plates were centrifuged at 1,200 rpm for 10 min, followed by the addition of 1% methylcellulose overlaid in each well. Plates were incubated at 37 °C with 5% CO2 for 3 days. After that, cells were fixed with ice-cold 80% acetone (Sigma) and washed three times with PBS supplemented with 0.05% Tween-20 (PBST). Fixed cells were blocked with blocking buffer (Odyssey, Cat. 927–40000) for 30 min, followed by 1-hor incubation with a rabbit anti-F monoclonal antibody (Sino Biologics) and washed with PBST before anti-rabbit IgG Alex488 conjugated secondary antibodies (Invitrogen) was added (1:500 diluted). Unbound secondary antibodies were washed off following 1 hour of incubation. Plates were read and counted by EnSight (PerkinElmer). The IC50 was calculated from a four-parameter non-linear fitting algorithm using Graph Pad Prism software. Data was presented as mean values ± SD of three replicates.

### Air-liquid interface cultures of HBECs

Primary human bronchial epithelial cells (HBECs) from healthy donors were obtained from Lonza (CC-2540). ALI cultures were performed by following the manufacturer’s protocol. Briefly, HBECs were cultured and expanded twice with PneumaCult™-EX Plus Medium (STEMCELL Technologies™, Cat. 05040) in T75 flasks. Afterward, HBECs were seeded on the permeable insert of 96-well Transwell plates (Corning, Cat. 7369) at a density of 15,000 cells per well. The cells were cultured in the expansion media on both apical and basal side of Transwell for 5 days. Then, the expansion medium was removed from both apical and basal side, and the differentiation medium (PneumaCult-ALI Plus Medium, STEMCELL Technologies™, Cat. 05001) was added only in the basal side of Transwell. Differentiation into a pseudostratified mucociliary epithelium was achieved after 3 weeks. Cells were cultured at an ALI condition for three weeks until used for qPCR analysis or transcytosis evaluation. During the differentiation period, the differentiation media in the basal side was exchanged to fresh media every 2–3 days, and an apical wash with the differentiation media was performed once per week.

For transcytosis assessment of antibodies in ALI cultures, tested antibodies were 4-fold serially diluted in a 6-point titration and added in the basal chambers of Transwell. After 24 or 48 hours, fresh media was added to the apical side to hydrate the mucus for 30 minutes and then 100 μL of the apical and basal media was collected for antibody quantification using the electrochemiluminescence method which was described in the Method of Transcytosis activity in MDCK cells.

### Quantitative polymerase chain reaction

HBECs were harvested from the transwell insert filter of ALI cultures at two time points, pre- and post-airlift. Total RNA was extracted using Zymo Quick-RNA extraction kit (Cat. R1054) per the manufacturer’s instructions. Reverse transcription was performed on extracted RNA samples using a Superscript IV VILO kit (Invitrogen, Cat. 11756050), followed by quantitative polymerase chain reaction (qPCR) using TaqMan Universal PCR Master Mix (Invitrogen Cat. 43-044-37) and TaqMan Gene Expression Assay kits. The reaction was run in an Applied Biosystems QuantStudio 7 Flex System (Thermo Fisher Scientific). To characterize the cell differentiation of ALI cultures, TaqMan Gene Expression Assays (Thermo Scientific) were used to target the following proteins in cells collected from human ALI cultures: p63 (Hs00978340_m1), ITGA6 (Hs01041011_m1), FOXA3 (Hs00270130_m1), Muc5AC (Hs01365601_m1), MUC5B (Hs00861588_m1), FOXJ1 (Hs00230964_m1), DNAI2 (Hs01001544_m1) and beta-actin (Hs99999903_m1). TaqMan Gene Expression Assay featured for pIgR (Hs00922561_m1) was used in cells collected from ALI cultures and MDCK cells which were included as controls. Comparative cycle threshold (Ct) values were determined using the method described by Schmittgen and Livakusing normalization to the housekeeping gene beta-actin (57).

### Pharmacokinetic analysis

Cotton rats (*Sigmodon hispidus*) were obtained from Envigo (Indianapolis, IN, USA). Wild type CD1 mice were purchased from Charles River Laboratories (Hollister, CA, USA). Three female cotton rats (4–7 weeks old) per sample group were injected intramuscularly with one dose of RSV/pIgR bsAb or anti-RSV IgG, 3 mg/kg body weight. Animals were administered 100 µL of antibody into the left quadriceps on day 0 under isoflurane anesthesia. Serum samples were collected for determining serum antibody concentrations at a series of time points: 0 hour, 1 hour, 6 hours, day 1, day 2, day 4 and day 10. To evaluate anti-pIgR V_H_H-hFc molecules in mice, three wildtype CD1 mice (6–8 weeks old) per group were dosed intravenously with anti-pIgR V_H_H-hFc molecules at 3 mg/kg body weight via the saphenous vein. Serum was collected at various time-points, ranging from 5 minutes to 14 days post antibody administration. For both PK studies, serum antibody concentrations were measured by the electrochemiluminescence method. The animal studies were approved by Merck Sharp & Dohme LLC, a subsidiary of Merck & Co., Inc., Rahway, NJ, USA, Institutional Animal Care and Use Committee and conducted in accordance with animal care guidelines.

### Statistical analysis

Data are presented as mean ± standard deviation and were analyzed using GraphPad Prism version 10.2.2 for Windows (GraphPad Software). Statistical correlation was performed using Pearson correlation analysis to compute the correlation coefficients (*r*) and p values. Statistical significance was determined using Ordinary one-way analysis of variance (ANOVA) followed by Tukey’s test for multiple comparisons, where appropriate. A *p* value lower than 0.05 was considered statistically significant.

## Data Availability

The original contributions presented in the study are included in the article/**Supplementary Material**. Further inquiries can be directed to the corresponding author.
