# Supplementary material for: Unlocking binding properties of single-domain antibodies targeting the polymeric immunoglobulin receptor to enhance mucosal enrichment of IgG against respiratory syncytial virus
Source: Front Immunol. 2026 Feb 9;16:1739562. doi: 10.3389/fimmu.2025.1739562 (PMC12926710; doi:10.3389/fimmu.2025.1739562)
Supplement: Supplementary file 1 [file SupplementaryFile1.docx]

***Supplementary Materials***

**Unlocking Binding Properties of Single-Domain Antibodies Targeting the Polymeric Immunoglobulin Receptor to Enhance Mucosal Enrichment of IgG Against Respiratory Syncytial Virus**

Jia Liu^1*^, Jiwon Jung^1^, Siqun Zhou^2^, Shi-Juan Chen^1^, Jiping Huang^1^, Yinyan Tang^1^, Karin Vroom^1^, Soha Motlagh^1^, Daoyang Chen^1^, Huong Trinh^3^, Peter Worthington^3^, Daphne Y. Ma^4^, Zhiyun Wen^4^, Bin Luo^4^, Daniela Bumbaca Yadav^2^, Jia Yao Phuah^4^, Zhifeng Chen^4^, Kalpit A. Vora^4^, Masahisa Handa^1^

^1^Department of Discovery Biologics, ^2^Department of Pharmacokinetics, Dynamics, Metabolism and Bioanalytics, ^3^Department of Quantitative Biosciences, ^4^Department of Infectious Diseases and Vaccines, Merck Research Laboratories (MRL), Merck & Co., Inc., Rahway, NJ, USA

*** Correspondence:**

**Jia Liu**

[**Jia.liu8@merck.com**](mailto:Jia.liu8@merck.com) Merck & Co., Inc., 213 East Grand Ave. South San Francisco California 94080, USA

**Keywords**: polymeric immunoglobulin receptor (pIgR); single-domain antibody (V_H_H); pH-dependent binding; respiratory syncytial virus (RSV); targeted mucosal delivery; bi-specific antibody.

**Table S1. Binding kinetics, cell binding EC_50_ to cpIgR-expressing MDCK cells, domain mapping, epitope binning, cross-reactivity to hpIgR and mpIgR, and transcytosis activity in cpIgR-expressing MDCK cells. (Data grouped by binding domains and subsequently sorted by K_D_ values in descending order.)**

| Sample Clone ID | cpIgR | | | Binding to cpIgR-MDCK cells | Binding ECD domain | Epitope binning | Cross-reactivity | | Transcytosed antibody concentrations in cpIgR-MDCK cells (nM ± SD) |
| --- | --- | --- | --- | --- | --- | --- | --- | --- | --- |
|  | *k_on_* (1/Ms) | *k_off_* (1/s) | K_D_ (M) | EC50 (nM) |  |  | hpIgR reactivity | mpIgR reactivity |  |
| VHH19 | 3.7E+04 | 8.0E-04 | 2.2E-08 | 0.89 | D1 | bin 1 | Yes | Yes | 1.50±0.24 |
| VHH35 | 5.6E+04 | 1.0E-03 | 1.8E-08 | 0.82 | D1 | bin 1 | Yes | Yes | 1.76±0.15 |
| VHH42 | 7.5E+04 | 1.0E-03 | 1.4E-08 | 1.97 | D1 | bin 1 | Yes | Yes | 1.85±0.07 |
| VHH36 | 2.8E+04 | 3.2E-04 | 1.1E-08 | 1.35 | D1 | bin 1 | Yes | Yes | 1.50±0.15 |
| VHH43 | 7.6E+04 | 7.0E-04 | 9.2E-09 | 1.33 | D1 | bin 1 | Yes | Yes | 1.76±0.04 |
| VHH18 | 3.3E+04 | 3.0E-04 | 9.0E-09 | 0.37 | D1 | bin 1 | Yes | Yes | 1.51±0.27 |
| VHH25 | 1.1E+05 | 9.2E-04 | 8.7E-09 | 1.85 | D1 | bin 1 | Yes | Yes | 1.80±0.01 |
| VHH40 | 4.1E+04 | 3.5E-04 | 8.4E-09 | 1.33 | D1 | bin 1 | Yes | Yes | 1.27±0.12 |
| VHH6 | 7.7E+04 | 6.4E-04 | 8.3E-09 | 0.66 | D1 | bin 1 | Yes | Yes | 1.30±0.28 |
| VHH11 | 6.6E+04 | 4.4E-04 | 6.7E-09 | 1.37 | D1 | bin 1 | Yes | Yes | 1.41±0.02 |
| VHH3 | 8.1E+04 | 5.3E-04 | 6.5E-09 | 16.36 | D1 | bin 1 | Yes | Yes | 1.51±0.23 |
| VHH14 | 3.7E+04 | 2.4E-04 | 6.5E-09 | 39.28 | D1 | bin 1 | Yes | Yes | 1.13±0.28 |
| VHH23 | 3.2E+04 | 2.1E-04 | 6.5E-09 | 4.85 | D1 | bin 1 | Yes | Yes | 1.15±0.05 |
| VHH30 | 5.1E+04 | 3.0E-04 | 6.0E-09 | 1.05 | D1 | bin 1 | Yes | Yes | 1.41±0.31 |
| VHH38 | 8.2E+04 | 4.4E-04 | 5.4E-09 | 0.35 | D1 | bin 1 | Yes | Yes | 1.22±0.15 |
| VHH26 | 6.9E+04 | 3.7E-04 | 5.4E-09 | 0.62 | D1 | bin 1 | Yes | Yes | 1.42±0.22 |
| VHH44 | 6.5E+04 | 3.3E-04 | 5.1E-09 | 2.32 | D1 | bin 1 | Yes | Yes | 1.77±0.46 |
| VHH33 | 3.5E+04 | 1.5E-04 | 4.3E-09 | 2.07 | D1 | bin 1 | Yes | Yes | 1.85±0.10 |
| VHH20 | 1.4E+04 | 5.8E-05 | 4.1E-09 | 3.77 | D1 | bin 1 | Yes | Yes | 1.12±0.26 |
| VHH10 | 6.1E+04 | 1.9E-04 | 3.2E-09 | 1.00 | D1 | bin 1 | Yes | Yes | 1.09±0.07 |
| VHH45 | 4.8E+04 | 1.4E-04 | 3.0E-09 | 0.46 | D1 | bin 1 | Yes | Yes | 1.26±0.08 |
| VHH29 | 6.8E+04 | 2.0E-04 | 3.0E-09 | 0.93 | D1 | bin 1 | Yes | Yes | 1.30±0.11 |
| VHH27 | 4.1E+04 | 1.2E-04 | 2.8E-09 | 1.12 | D1 | bin 1 | Yes | Yes | 1.49±0.05 |
| VHH32 | 4.3E+04 | 1.0E-04 | 2.4E-09 | 0.52 | D1 | bin 1 | Yes | Yes | 1.60±0.07 |
| VHH1 | 5.1E+04 | 1.1E-04 | 2.2E-09 | 2.61 | D1 | bin 1 | Yes | Yes | 2.24±1.08 |
| VHH2 | 6.4E+04 | 1.1E-04 | 1.7E-09 | 2.05 | D1 | bin 1 | Yes | Yes | 1.04±0.12 |
| VHH9 | 4.0E+04 | 6.4E-05 | 1.6E-09 | 0.78 | D1 | bin 1 | Yes | Yes | 1.29±0.06 |
| VHH13 | 3.4E+04 | 3.7E-05 | 1.1E-09 | 2.83 | D1 | bin 1 | Yes | Yes | 1.07±0.19 |
| VHH21 | 5.6E+04 | 4.9E-05 | 8.8E-10 | 0.63 | D1 | bin 1 | Yes | Yes | 0.93±0.12 |
| VHH28 | 5.1E+04 | 4.4E-05 | 8.6E-10 | 0.58 | D1 | bin 1 | Yes | Yes | 1.23±0.10 |
| VHH31 | 5.3E+04 | 4.5E-05 | 8.4E-10 | 0.94 | D1 | bin 1 | Yes | Yes | 1.29±0.10 |
| VHH41 | 6.5E+04 | 4.7E-05 | 7.2E-10 | 0.78 | D1 | bin 1 | Yes | Yes | 1.75±0.14 |
| VHH15 | 5.2E+04 | 1.2E-05 | 2.4E-10 | 0.99 | D1 | bin 1 | Yes | Yes | 1.10±0.19 |
| VHH5 | 5.3E+04 | 4.6E-06 | 8.6E-11 | 0.86 | D1 | bin 1 | Yes | Yes | 1.04±0.18 |
| VHH12 | 7.2E+04 | 4.6E-06 | 6.4E-11 | 1.61 | D1 | bin 1 | Yes | Yes | 1.04±0.13 |
| VHH4 | 4.8E+04 | 7.5E-07 | 1.6E-11 | 0.85 | D1 | bin 1 | Yes | Yes | 1.07±0.20 |
| VHH16 | 2.6E+05 | 5.8E-02 | 2.2E-07 | NB | D2 | ND | No | Yes | 0.06±0.01 |
| VHH37 | 3.1E+05 | 1.4E-02 | 4.5E-08 | 0.41 | D2 | bin 3 | No | Yes | 1.16±0.12 |
| VHH8 | 1.5E+05 | 2.8E-03 | 1.8E-08 | 0.30 | D2 | bin 2 | No | Yes | 1.33±0.27 |
| VHH24 | 1.9E+09 | 1.2E+01 | 6.6E-09 | NB | D2 | ND | No | Yes | 0.12±0.05 |
| VHH39 | 2.2E+05 | 3.2E-04 | 1.4E-09 | 0.64 | D2 | bin 2 | No | Yes | 0.94±0.09 |
| VHH17 | 1.1E+05 | 2.5E-04 | 2.2E-09 | 0.81 | D3 | bin 6 | No | Yes | 1.65±0.41 |
| VHH7 | 2.6E+05 | 1.3E-04 | 5.1E-10 | 1.45 | D3 | bin 4 | Yes | Yes | 0.95±0.17 |
| VHH34 | 1.0E+05 | 3.7E-03 | 3.6E-08 | 0.16 | D4-5 | bin 5 | No | Yes | 2.23±0.28 |
| VHH22 | 2.2E+05 | 1.1E-03 | 4.8E-09 | 0.18 | D4-5 | bin 5 | No | No | 1.14±0.15 |

NB indicates “not bind”; ND indicates “not detect”.

**Table S2. Construct sequences of full-length ECD of cpIgR, hpIgR, mpIgR and truncated domain variants of cpIgR**

| **Construct Name** | **Residues** | **Signal Peptide Sequence (SLAM)** | **Mature Protein Sequence** |
| --- | --- | --- | --- |
| **Human pIgR D1-5 (full length ECD domains)** | SP(SLAM)-1-547-linker-Avi-His | MDPKGLLSLTFVLFLSLAFG | KSPIFGPEEVNSVEGNSVSITCYYPPTSVNRHTRKYWCRQGARGGCITLISSEGYVSSKYAGRANLTNFPENGTFVVNIAQLSQDDSGRYKCGLGINSRGLSFDVSLEVSQGPGLLNDTKVYTVDLGRTVTINCPFKTENAQKRKSLYKQIGLYPVLVIDSSGYVNPNYTGRIRLDIQGTGQLLFSVVINQLRLSDAGQYLCQAGDDSNSNKKNADLQVLKPEPELVYEDLRGSVTFHCALGPEVANVAKFLCRQSSGENCDVVVNTLGKRAPAFEGRILLNPQDKDGSFSVVITGLRKEDAGRYLCGAHSDGQLQEGSPIQAWQLFVNEESTIPRSPTVVKGVAGGSVAVLCPYNRKESKSIKYWCLWEGAQNGRCPLLVDSEGWVKAQYEGRLSLLEEPGNGTFTVILNQLTSRDAGFYWCLTNGDTLWRTTVEIKIIEGEPNLKVPGNVTAVLGETLKVPCHFPCKFSSYEKYWCKWNNTGCQALPSQDEGPSKAFVNCDENSRLVSLTLNLVTRADEGWYWCGVKQGHFYGETAAVYVAVEERGSGLNDIFEAQKIEWHEHHHHHH |
| **Mouse pIgR D1-5 (full length ECD domains)** | SP(SLAM)-1-549-linker-Avi-His | MDPKGLLSLTFVLFLSLAFG | KSPIFGPQEVSSIEGDSVSITCYYPDTSVNRHTRKYWCRQGASGMCTTLISSNGYLSKEYSGRANLINFPENNTFVINIEQLTQDDTGSYKCGLGTSNRGLSFDVSLEVSQVPELPSDTHVYTKDIGRNVTIECPFKRENAPSKKSLCKKTNQSCELVIDSTEKVNPSYIGRAKLFMKGTDLTVFYVNISHLTHNDAGLYICQAGEGPSADKKNVDLQVLAPEPELLYKDLRSSVTFECDLGREVANEAKYLCRMNKETCDVIINTLGKRDPDFEGRILITPKDDNGRFSVLITGLRKEDAGHYQCGAHSSGLPQEGWPIQTWQLFVNEESTIPNRRSVVKGVTGGSVAIACPYNPKESSSLKYWCRWEGDGNGHCPVLVGTQAQVQEEYEGRLALFDQPGNGTYTVILNQLTTEDAGFYWCLTNGDSRWRTTIELQVAEATREPNLEVTPQNATAVLGETFTVSCHYPCKFYSQEKYWCKWSNKGCHILPSHDEGARQSSVSCDQSSQLVSMTLNPVSKEDEGWYWCGVKQGQTYGETTAIYIAVEERGSGLNDIFEAQKIEWHEHHHHHH |
| **Cotton rat pIgR D1-5 (full length ECD domains)** | SP(SLAM)-1-547-linker-Avi-His | MDPKGLLSLTFVLFLSLAFG | KSPIFGPQDVSSVVGNSVSITCYYPDTSVNRHTRKYWCRQGASGLCTTLISSQGFVSKEYAGRASIINFPGNNTFVINIAQLTQSDTGSYKCGLGTSNQGLFFDVSLEVSQVPELPADTHVYTKDLGRNVIIDCPFKLENAHSKKSLCKKTGQTCELVIDSVEYVNPKYKDRIKLFTKGTSKEKFSVNITNLRLSDAGLYVCQAGEDPKADKKNVDLQVLKPEPELVYGDLRASVTFDCALGREVANEAKYLCRMNKDKCDVVINTLGKRDPSFEGRILLTPKDKNGRFSILITGLRREDAGYYLCGAHSSGLPQEGWPIQSWHLFVNEESTIPNSRSVVKGVTGGSVAITCPYNPKDNNSLKYWCHWDENGHCPILVDSQGLKEEQYVGRLALYDQPGNGIYTVILNQLTTKDAGFYWCLTNGDSRWRTTIELQVAEATGKPNLEVTPQNVNAVLGESVEISCHYPCKFYSHEKYWCKWSSQGCHVLPSQDKGARQPSVSCDQNNQVISITLNPVKKEDEGWYWCGVKQGQVYGETTAIYVAVGERGSGLNDIFEAQKIEWHEHHHHHH |
| **Cotton rat pIgR D1** | SP(SLAM)-1-110-linker-Avi-His | MDPKGLLSLTFVLFLSLAFG | KSPIFGPQDVSSVVGNSVSITCYYPDTSVNRHTRKYWCRQGASGLCTTLISSQGFVSKEYAGRASIINFPGNNTFVINIAQLTQSDTGSYKCGLGTSNQGLFFDVSLEVSGSGLNDIFEAQKIEWHEHHHHHH |
| **Cotton rat pIgR D1-2** | SP(SLAM)-1-218-linker-Avi-His | MDPKGLLSLTFVLFLSLAFG | KSPIFGPQDVSSVVGNSVSITCYYPDTSVNRHTRKYWCRQGASGLCTTLISSQGFVSKEYAGRASIINFPGNNTFVINIAQLTQSDTGSYKCGLGTSNQGLFFDVSLEVSQVPELPADTHVYTKDLGRNVIIDCPFKLENAHSKKSLCKKTGQTCELVIDSVEYVNPKYKDRIKLFTKGTSKEKFSVNITNLRLSDAGLYVCQAGEDPKADKKNVDLQGSGLNDIFEAQKIEWHEHHHHHH |
| **Cotton rat pIgR D1-3** | SP(SLAM)-1-328-linker-Avi-His | MDPKGLLSLTFVLFLSLAFG | KSPIFGPQDVSSVVGNSVSITCYYPDTSVNRHTRKYWCRQGASGLCTTLISSQGFVSKEYAGRASIINFPGNNTFVINIAQLTQSDTGSYKCGLGTSNQGLFFDVSLEVSQVPELPADTHVYTKDLGRNVIIDCPFKLENAHSKKSLCKKTGQTCELVIDSVEYVNPKYKDRIKLFTKGTSKEKFSVNITNLRLSDAGLYVCQAGEDPKADKKNVDLQVLKPEPELVYGDLRASVTFDCALGREVANEAKYLCRMNKDKCDVVINTLGKRDPSFEGRILLTPKDKNGRFSILITGLRREDAGYYLCGAHSSGLPQEGWPIQSWHLFVNGSGLNDIFEAQKIEWHEHHHHHH |
| **Cotton rat pIgR D4-5** | SP(SLAM)-338-547-linker-Avi-His | MDPKGLLSLTFVLFLSLAFG | SVVKGVTGGSVAITCPYNPKDNNSLKYWCHWDENGHCPILVDSQGLKEEQYVGRLALYDQPGNGIYTVILNQLTTKDAGFYWCLTNGDSRWRTTIELQVAEATGKPNLEVTPQNVNAVLGESVEISCHYPCKFYSHEKYWCKWSSQGCHVLPSQDKGARQPSVSCDQNNQVISITLNPVKKEDEGWYWCGVKQGQVYGETTAIYVAVGERGSGLNDIFEAQKIEWHEHHHHHH |

**Table S3.** **Ratios of affinity, dissociation rate, and association rate at pH 5.5 versus pH 7.6 for anti-pIgR V_H_H-hFc molecules displaying intermediate affinities (1-10 nM).**

| Sample Clone ID | Transcytosed antibody concentrations in cpIgR-MDCK cells (nM ± SD) | K_D_ at pH 7.6 (M) | Ratios (pH5.5/pH7.6) | | |
| --- | --- | --- | --- | --- | --- |
|  |  |  | K_D_ ratio | k*_off_* ratio | k*_on_* ratio |
| VHH25 | 1.80±0.01 | 8.7E-09 | 3.5 | 1.7 | 0.5 |
| VHH43 | 1.76±0.04 | 9.2E-09 | 0.0 | 0.1 | 4.4 |
| VHH3 | 1.51±0.23 | 6.5E-09 | 3.4 | 5.9 | 1.8 |
| VHH18 | 1.51±0.27 | 9.0E-09 | 1.2 | 2.6 | 2.2 |
| VHH27 | 1.49±0.05 | 2.8E-09 | 15.3 | 23.9 | 1.6 |
| VHH30 | 1.41±0.31 | 6.0E-09 | 2.3 | 11.8 | 5.1 |
| VHH11 | 1.41±0.02 | 6.7E-09 | 4.0 | 6.8 | 1.7 |
| VHH29 | 1.30±0.11 | 3.0E-09 | 9.8 | 6.3 | 0.6 |
| VHH6 | 1.30±0.28 | 8.3E-09 | 4.3 | 3.0 | 0.7 |
| VHH9 | 1.29±0.06 | 1.6E-09 | 14.0 | 19.3 | 1.4 |
| VHH40 | 1.27±0.12 | 8.4E-09 | 21.1 | 17.9 | 0.9 |
| VHH45 | 1.26±0.08 | 3.0E-09 | 2.4 | 4.1 | 1.7 |
| VHH38 | 1.22±0.15 | 5.4E-09 | 4.2 | 1.8 | 0.4 |
| VHH23 | 1.15±0.05 | 6.5E-09 | 1.0 | 1.9 | 1.9 |
| VHH14 | 1.13±0.28 | 6.5E-09 | 3.4 | 4.0 | 1.2 |
| VHH20 | 1.12±0.26 | 4.1E-09 | 4.6 | 18.5 | 4.0 |
| VHH10 | 1.09±0.07 | 3.2E-09 | 8.2 | 10.7 | 1.3 |
| VHH13 | 1.07±0.19 | 1.1E-09 | 7.7 | 16.8 | 2.2 |
| VHH2 | 1.04±0.12 | 1.7E-09 | 4.1 | 4.5 | 1.1 |

**Table S4-1.** **Characterization of RSV/pIgR bsAb (VHH1) using SEC and CE-SDS**

| **Sample Name** | **SEC** | | | | **CE-SDS_Non-Reduced** | | | **CE-SDS_Reduced** | | |
| --- | --- | --- | --- | --- | --- | --- | --- | --- | --- | --- |
|  | %HMW | %Main | %LMW | Retention Time (min) | %HMW | %Main | %LMW | %LC | %HC | %Others |
| RSV/pIgR bsAb (VHH1) | 2.4 | 97.60 | 0 | 2.62 | 0 | 99.61 | 0.39 | 27.38 | 72.25 | 0.37 |

**Table S4-2.** **Mass spectrometric analysis of RSV/pIgR bsAb (VHH1)**

| **Sample Name** | **Predicted Mass (Da)** | | | **Observed Mass (Da)** | | | | | | **Other Peak** |
| --- | --- | --- | --- | --- | --- | --- | --- | --- | --- | --- |
|  | Intact | LC | HC | Intact | Error | LC | Error | HC | Error |  |
| RSV/pIgR  bsAb (VHH1) | 176418 | 22923 | 65304 | 176420 | 2 | 22920 | -3 | 65296 | -8 | None |

HMW: high molecular weight; LMW: low molecular weight; LC: light chain; HC: heavy chain.

**Figure S1. Association rates (*k_on_)* at pH7.6 (x-axis) are plotted vs. transcytosis activity (y-axis) for 36 anti-pIgR V_H_H-hFc molecules.** Each data point represents the mean value of three replicates for an individual molecule, with standard deviation bars indicating variability. The Pearson Correlation coefficient (*r*) and significance were calculated and indicated in the graph.


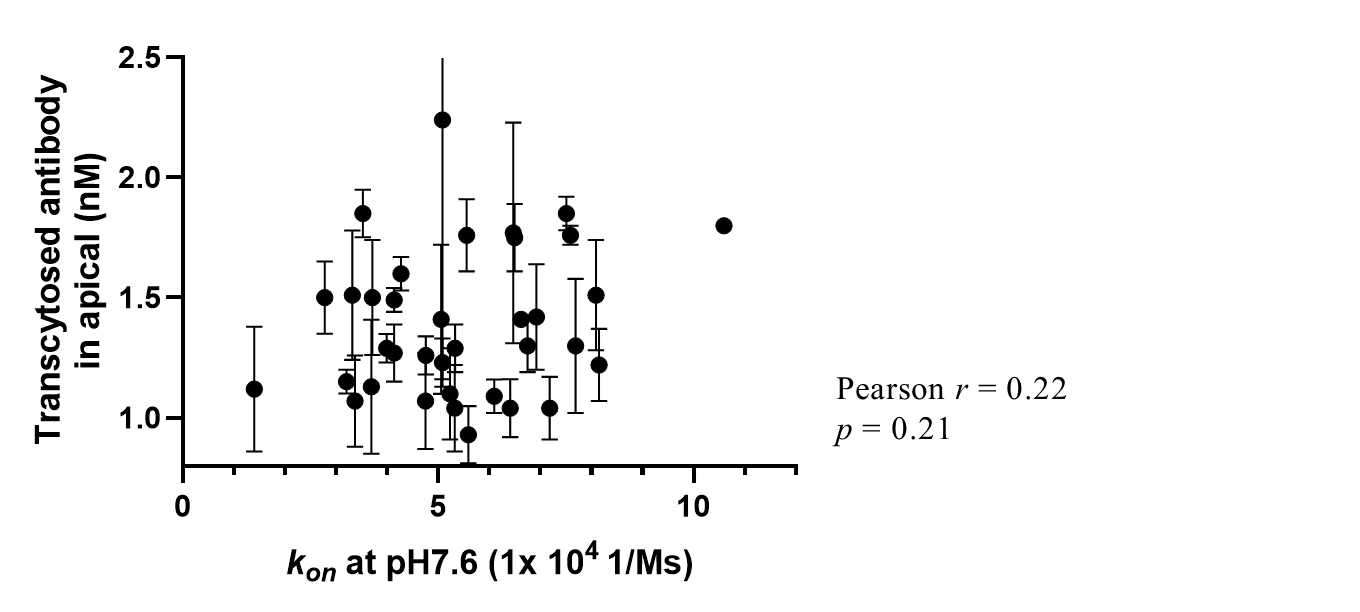


**Figure S2 and Table S5. Plasma PK profile of antibodies in cotton rats (A) and mice (B).**

A.


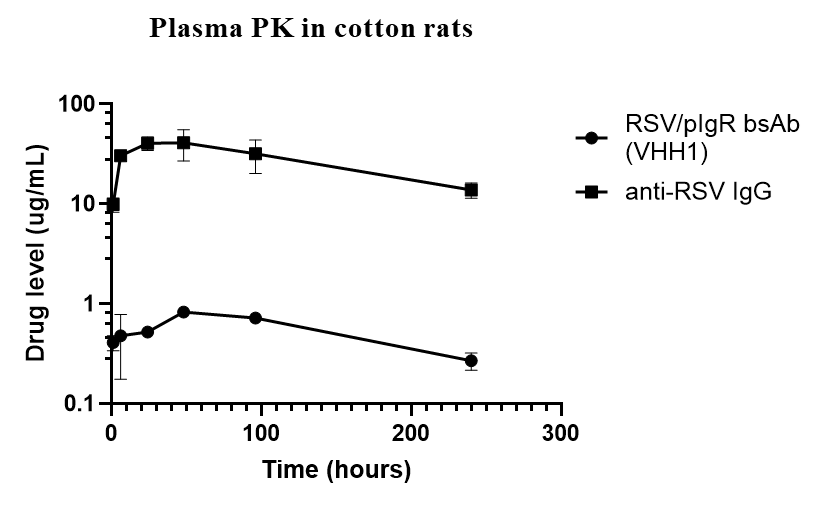


B.


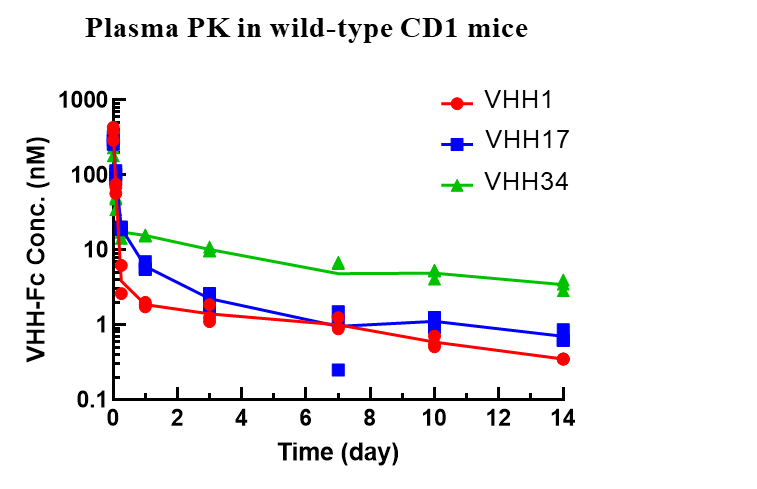


Table S5. Plasma PK profile of selected anti-pIgR V_H_H-hFc in wild type CD1 mice

| Anti-pIgR VHH-hFc clone ID | Binding affinity to mouse pIgR  K_D_ (nM) | CL  (mL/d/kg) | Vss  (mL/kg) | t1/2  (d) |
| --- | --- | --- | --- | --- |
| VHH1 | 0.56 | 1060 ± 153 | 3830 ± 1040 | 4.92 ± 0.96 |
| VHH17 | 38 | 661 ± 76.9 | 2580 ± 830 | 5.03 ± 2.81 |
| VHH34 | 11 | 269 ± 78.2 | 2340 ± 140 | 6.81 ± 2.54 |
